# Poly(trehalose methacrylate) as an Excipient for Insulin Stabilization: Mechanism and Safety

**DOI:** 10.1021/acsami.2c09301

**Published:** 2022-08-15

**Authors:** Madeline B. Gelb, Kathryn M. M. Messina, Daniele Vinciguerra, Jeong Hoon Ko, Jeffrey Collins, Mikayla Tamboline, Shili Xu, F. Javier Ibarrondo, Heather D. Maynard

**Affiliations:** † Department of Chemistry and Biochemistry and California NanoSystems Institute, University of California, Los Angeles, 607 Charles E. Young Drive East, Los Angeles, California 90095-1569, United States; ‡ Department of Molecular and Medical Pharmacology and Crump Institute for Molecular Imaging, David Geffen School of Medicine, University of California, Los Angeles, California 90095-1735, United States; § Division of Infectious Diseases, Department of Medicine, David Geffen School of Medicine at UCLA, Los Angeles, California 90095-1569, United States

**Keywords:** trehalose polymer, insulin stabilization, biodistribution, immune response

## Abstract

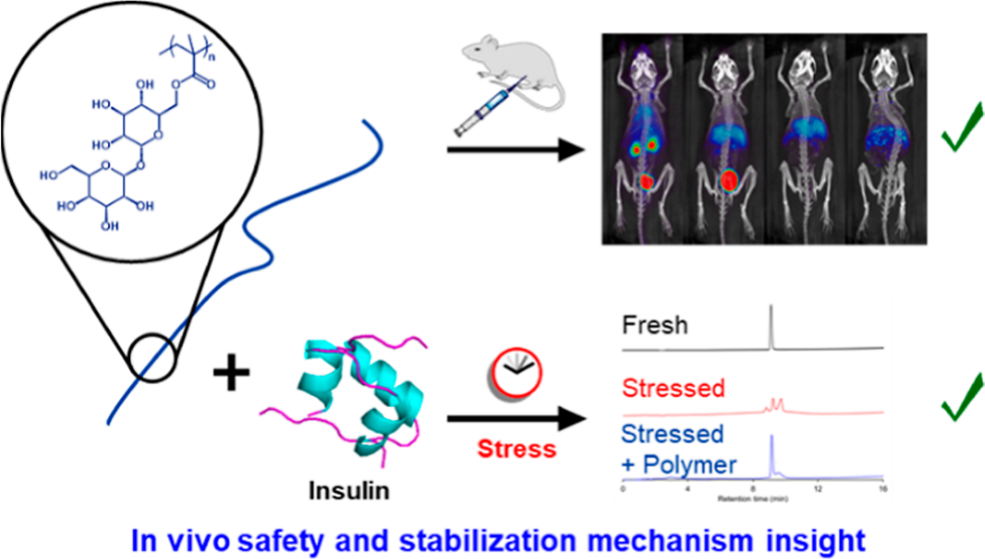

Insulin, the oldest U.S. Food and Drug Administration (FDA)-approved recombinant protein and a World Health Organization (WHO) essential medicine for treating diabetes globally, faces challenges due to its storage instability. One approach to stabilize insulin is the addition of poly(trehalose methacrylate) (pTrMA) as an excipient. The polymer increases the stability of the peptide to heat and mechanical agitation and has a low viscosity suitable for injection and pumps. However, the safety and stabilizing mechanism of pTrMA is not yet known and is required to understand the potential suitability of pTrMA as an insulin excipient. Herein is reported the immune response, biodistribution, and insulin plasma lifetime in mice, as well as investigation into insulin stabilization. pTrMA alone or formulated with ovalbumin did not elicit an antibody response over 3 weeks in mice, and there was no observable cytokine production in response to pTrMA. Micropositron emission tomography/microcomputer tomography of ^64^Cu-labeled pTrMA showed excretion of 78–79% ID/cc within 24 h and minimal liver accumulation at 6–8% ID/cc when studied out to 120 h. Further, the plasma lifetime of insulin in mice was not altered by added pTrMA. Formulating insulin with 2 mol equiv of pTrMA improved the stability of insulin to standard storage conditions: 46 weeks at 4 °C yielded 87.0% intact insulin with pTrMA present as compared to 7.8% intact insulin without the polymer. The mechanism by which pTrMA-stabilized insulin was revealed to be a combination of inhibiting deamidation of amino acid residues and preventing fibrillation, followed by aggregation of inactive and immunogenic amyloids all without complexing insulin into its hexameric state, which could delay the onset of insulin activity. Based on the data reported here, we suggest that pTrMA stabilizes insulin as an excipient without adverse effects in vivo and is promising to investigate further for the safe formulation of insulin.

## Introduction

Diabetes is a chronic metabolic disease that affects approximately 422 million people globally, making it one of the most pressing global health issues according to the World Health Organization (WHO) and Center for Disease Control.^1,2^ The large number of diabetic patients that rely on injections of insulin to prevent permanent damage caused by hyperglycemia including impaired kidney function and retinal damage has brought insulin onto the WHO model list of essential medicines.^3^ While insulin is effective for managing diabetes in many patients, there are still challenges in stabilizing insulin to environmental stressors that are encountered in manufacture, storage, and transportation, particularly because it is a worldwide disease. Insulin requires a cold chain before and after being delivered to patients, so increasing the stability of insulin formulations would minimize the costs of insulin lost due to poor storage and short expiration dates and allow delivery to places where the cold chain is limited.^4^

Insulin undergoes two main mechanisms of degradation through either physical or chemical means. Insulin degrades chemically through deamidation at Asn A21 under acidic conditions and at Asn B3 under neutral to basic conditions.^5^ Interestingly, deamidation does not appear to alter the activity of insulin in vivo. Physical degradation of insulin through aggregation and fibrillation is the major challenge associated with insulin instability and loss of activity.^6^ Insulin rapidly forms large fibrils upon exposure to heat and/or mechanical agitation.^7,8^ These aggregates can lead to a multitude of issues, including occlusion of needles and pumps, immunogenicity, and inadequate dosing.^8,9^ While there are many insulin analogues on the market that alter pharmacokinetics, these modifications are not intended to improve the storage stability. Insulin has long been formulated with Zn^2+^ in an effort to stabilize the protein; Zn^2+^ draws dimerized insulin together to form the more stable hexameric insulin.^10^ While this is a more stable configuration, insulin is only active in its monomeric state, so formulating with Zn^2+^ also slows down the onset of insulin activity. β-Cyclodextrin and cucurbit[7]uril have also been explored as stabilizing moieties for a variety of proteins including insulin.^11−13^ More recently, quite a few supramolecular materials have been devised by the Langer and Appel labs, and these largely polyethylene glycol (PEG) and polyacrylamide-based excipients have been shown to stabilize insulin in even its monomeric state.^14−17^ This supramolecular work, in particular, has focused on preventing the aggregation of insulin. Alternatively, small molecules and peptides have been identified and developed to inhibit insulin amyloidosis that would otherwise lead to the aggregation of the protein.^18−21^

**Figure 1:**
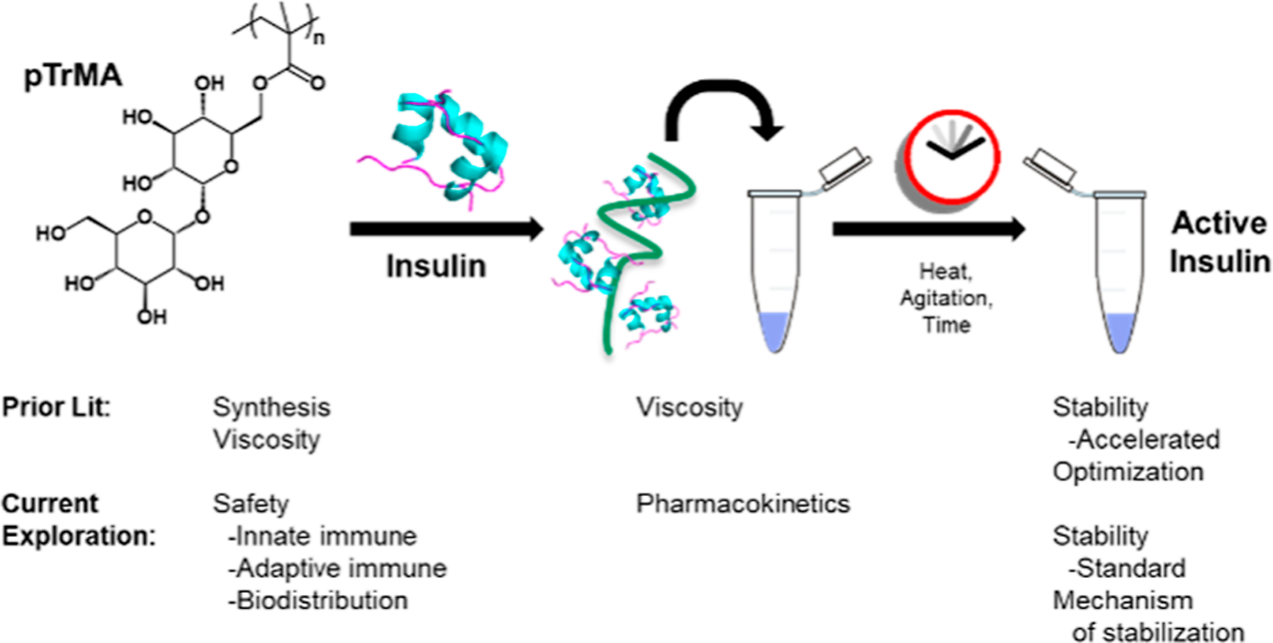
Prior and current pTrMA investigations.

Our group has developed polymers with side chains composed of the natural stabilizer trehalose. We have shown that these polymers stabilize antibodies, enzymes, growth factors, and hormones to environmental stressors.^22−31^ These polymers have also been shown to stabilize insulin. Studying insulin stabilization with a styrenyl ether-based trehalose polymer, we found that different regioisomers of the monomer all stabilized the peptide against heat and agitation, indicating that the trehalose itself is more important in stabilizing insulin than the site by which trehalose is attached to the backbone.^26^ Furthermore, we also found that covalently conjugating poly(trehalose methacrylate) (pTrMA) to insulin both prolonged the half-life in vivo and stabilized insulin against heat and mechanical agitation.^27,28^ We successfully optimized the pTrMA concentration and molecular weight (MW) for insulin stabilization against heat and agitation in order to reduce the amount of pTrMA in formulation for cost.^31^ Further, while the pTrMA did slightly increase the formulation viscosity from insulin alone, formulations were well below a known threshold for tolerable injections.^32^ This previous work has provided formulations of insulin but no understanding of the polymer safety or the mechanism by which pTrMA stabilizes insulin. Given the promising results obtained, we set out to investigate not only the safety profile of pTrMA through immune and biodistribution studies but also the mechanism by which the polymer stabilizes insulin. In addition, the long-term refrigeration stability of insulin with and without the polymer and the effect of the polymer excipient on the plasma lifetime of insulin are reported herein (Figure 1).

## Results

### Immunogenicity of pTrMA

**Figure 2:**
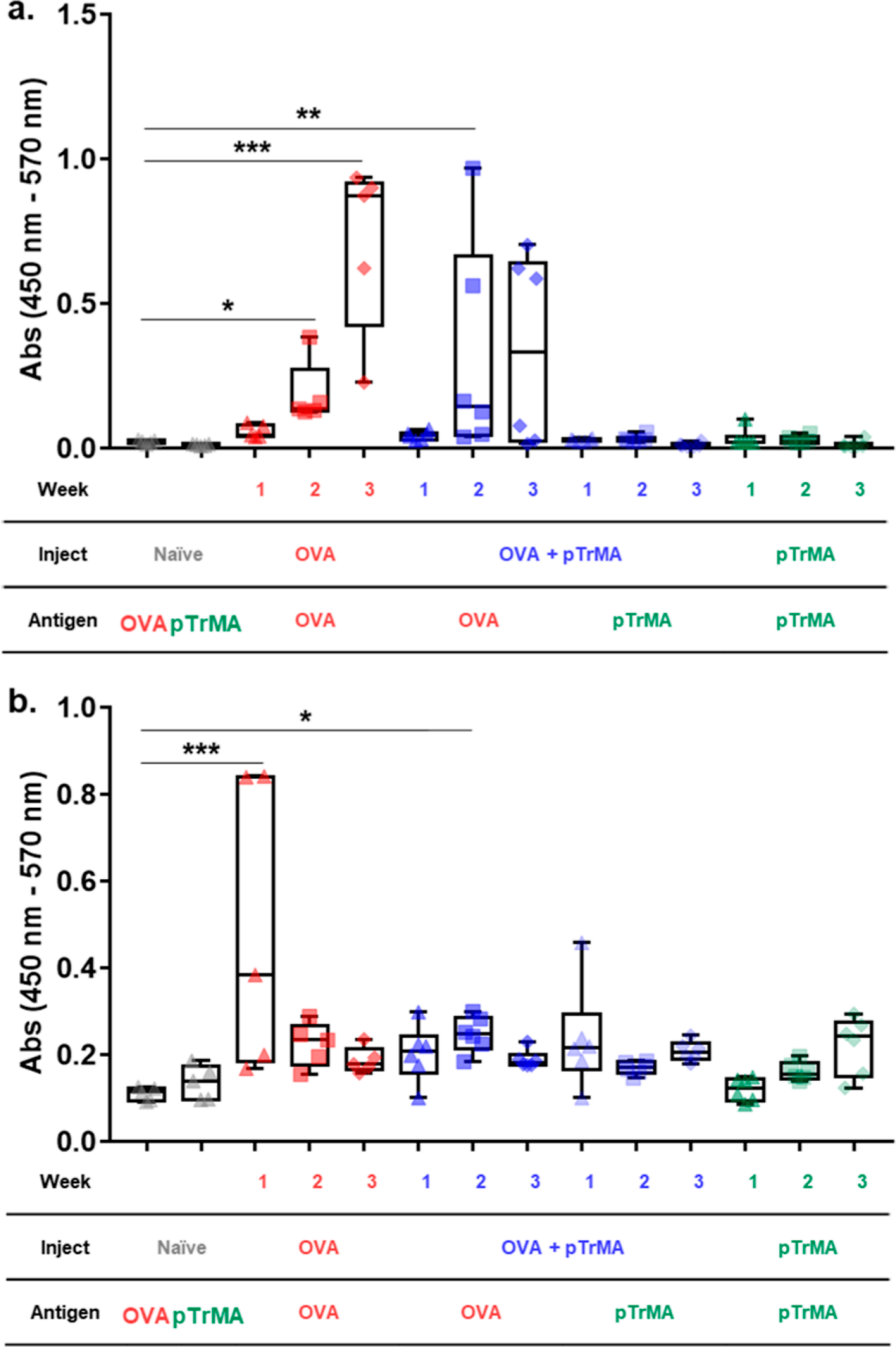
Antibody levels in mice specific to OVA or pTrMA (BSA–pTrMA conjugate) antigens over 3 weeks after i.p. injection of OVA (2 mg/kg), OVA + pTrMA (2 mg/kg, 10 wt %), or pTrMA (10 wt %) at weeks 0 and 2 (*n* = 5–6). Levels measured by immune ELISA for (a) IgG- and (b) IgM-specific antibody responses and compared to non-specific baseline antibody recognition in naïve mice (*p < 0.05, **p < 0.01, and ***p < 0.001 corresponding to the significant difference from the naïve control for the respective antigen).

To study the adaptive immune response in the form of antibody production, mice were challenged at weeks 0 and 2 with the model immunogenic protein ovalbumin (OVA, 2 mg/kg) as a positive control, pTrMA (10 wt % or 100 mg/mL), or both OVA + pTrMA (2 mg/kg OVA, 10 wt % pTrMA) via intraperitoneal (i.p.) injection. Serum was collected each week, and the immunoglobulin IgG and IgM antibodies specific to OVA or pTrMA were measured by ELISA. For ELISA, OVA or bovine serum albumin (BSA) conjugated with pTrMA (Figure S1) was adsorbed for 16–18 h on microplate wells to capture any antibodies in the serum. Serum from challenged mice and naïve mice as a negative control was measured for antibodies against the challenge antigen, OVA, or the BSA–pTrMA conjugate. Serum from the mice treated with OVA and pTrMA was tested against both OVA and the BSA–pTrMA conjugate. Testing against both antigens measured if the polymer increases the immunogenicity of OVA and, conversely, if the presence of an immunogenic protein, OVA, elicited an immunogenic response to the polymer (Figure S2). The resulting data indicated that only the OVA elicited an IgG response which, for OVA alone, increased in both weeks 2 and 3, and for OVA with trehalose polymer, increased from week 1 to week 2 but was not significantly different from naïve serum by week 3 (Figure 2a). While OVA did also elicit an IgM immune response, it tailed off quickly; with OVA alone, IgM was only elevated in the first week, whereas for OVA with the trehalose polymer, IgM was only elevated in the second week (Figure 2b). pTrMA did not induce any significant generation of polymer-specific IgG or IgM antibodies even in the presence of OVA.

**Figure 3:**
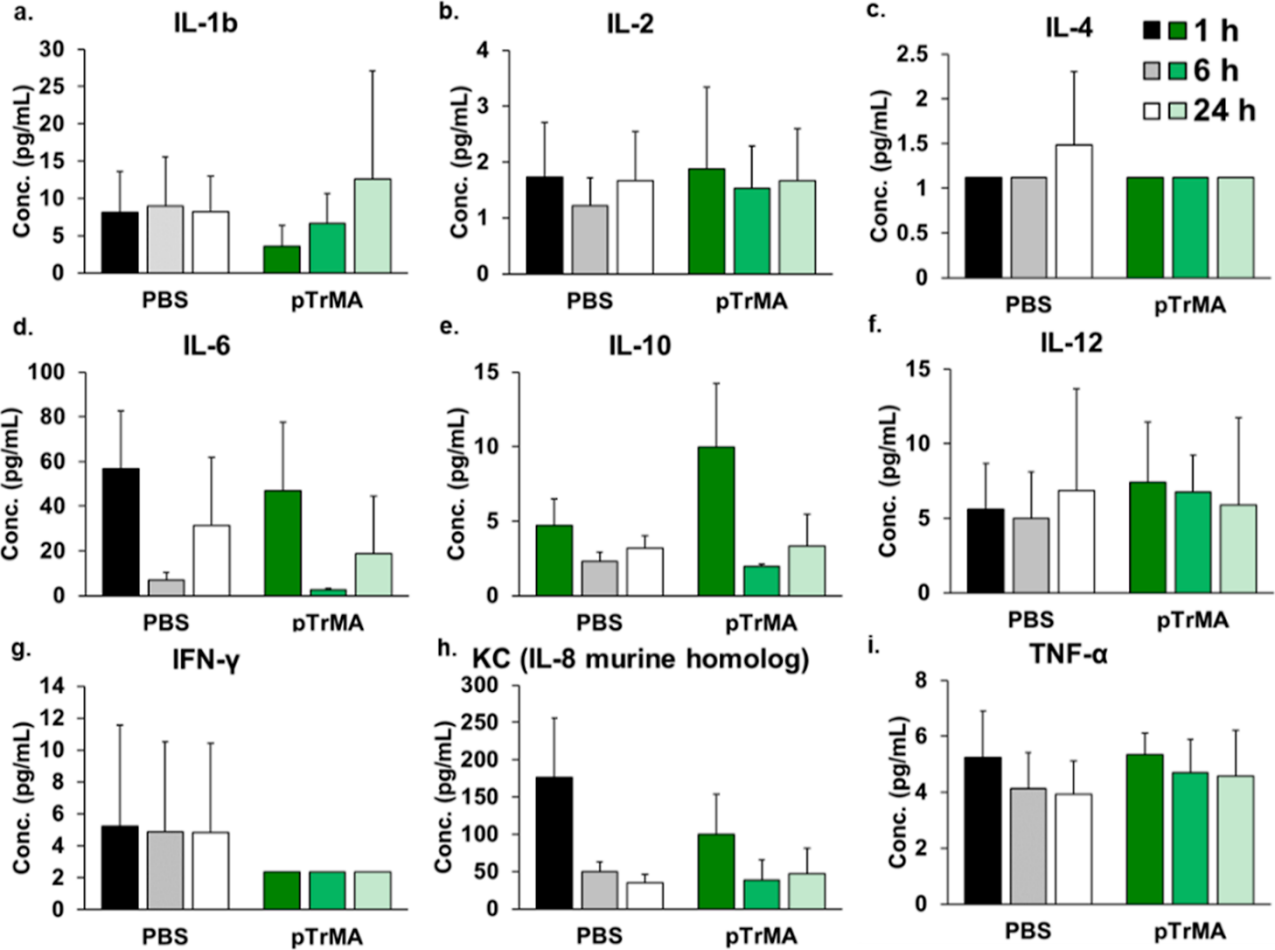
Plasma cytokine levels of (a) IL-1b, (b) IL-2, (c) IL-4, (d) IL-6, (e) IL-10, (f) IL-12, (g) IFN-γ, (h) KC, and (i) TNFα in mice (*n* = 5) at 1, 6, and 24 h post i.p. injection of PBS or pTrMA (10 mg/kg) measured by the Luminex xMAP immunoassay. Note that the PBS control data shown in this figure are reproduced with permission from ref (33). Copyright 2022 American Chemical Society.

For a full picture of immune response to pTrMA, the innate immune response was evaluated by measuring cytokine levels in mice. A library of pro-inflammatory [interleukin (IL)-1b, IL-2, IL-6, IL-12, KC (IL-8 analogue in mice), tumor necrosis factor α (TNF-α), and interferon-γ (IFN-γ)] and anti-inflammatory (IL-10 and IL-4) cytokines were detected by Luminex’s xMAP immunoassay, a multiplexed ELISA that measures multiple analytes at once. The cytokine levels were measured at 1, 6, and 24 after i.p. injection of phosphate-buffered saline (PBS) or pTrMA (10 mg/kg). As expected from the negative control, PBS, there was minimal production of any of the tested cytokines over the study (Figure 3). Cytokine levels in the mice exposed to pTrMA were comparable to the negative control group, indicating little innate immune response to the polymer. These cytokine measurements together with the antibody response results show pTrMA to not be immunogenic or inflammatory alone or in the presence of an immunogenic protein.

### Biodistribution, Excretion, and Inflammation with pTrMA-*co*-DOTA by Micropositron Emission Tomography and Microcomputer Tomography

**Figure 4:**
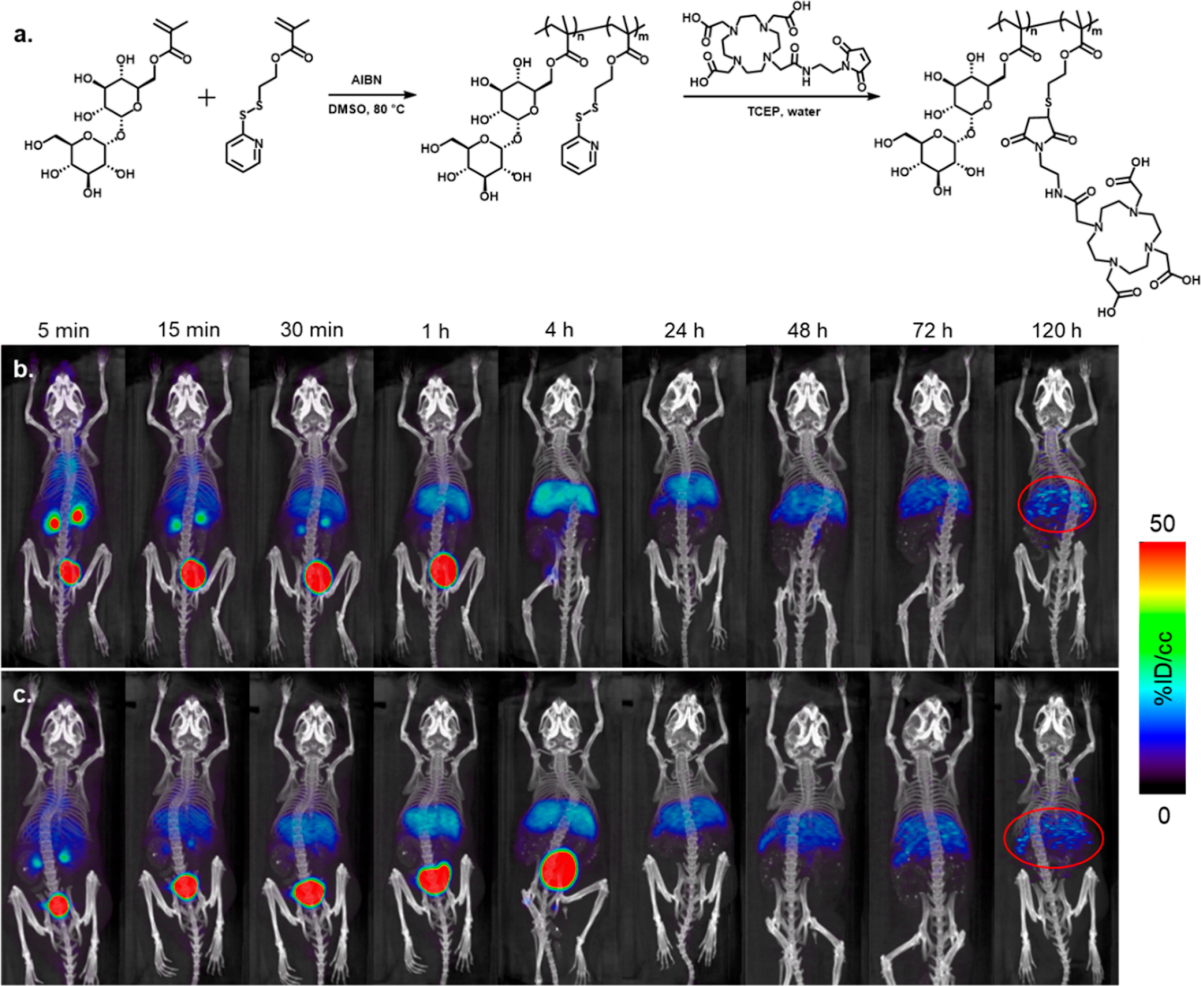
(a) Structure of pTrMA-*co*-DOTA and representative MIP μPET/μCT images from 5 min to 120 h post-injection of ^64^Cu-labeled pTrMA-*co*-DOTA (200 μCi) into (b) female (*n* = 4) and (c) male (*n* = 4) mice (dynamic scans 0–60 h and static scans for later time points). Circled region, liver.

pTrMA was synthesized as a random copolymer with a single unit of PDSMA on average per polymer chain (pTrMA-*co*-PDSMA) via free radical polymerization (Figure 4a). After the polymerization, the disulfide was reduced and reacted with the tetraaza 12-membered chelating ring DOTA via thiol-maleimide chemistry, forming pTrMA-*co*-DOTA. This modification successfully removed all PDSMA from the polymer; however, despite the attempts to improve modification, DOTA incorporation did not exceed 85%. Given that there was an average of a single PDSMA unit per polymer chain, 85% of the pTrMA polymers bore a single DOTA as a pendant side chain. This pTrMA-*co*-DOTA polymer (Figure 4a) could then chelate ^64^Cu with >99% efficiency by high-pressure liquid chromatography (HPLC).

Although the cytokine production studies indicated that pTrMA does not cause a systemic inflammatory response, to further examine the possibility of pTrMA causing localized inflammation in specifically inflamed organs, we conducted an experiment using ^18^F-fludeoxyglucose (FDG), a glucose analogue, and a radioactive tracer that is preferably taken up by inflamed tissues. Mice (female *n* = 4 and male *n* = 4) were i.v. injected with ^18^F-FDG as a baseline before injecting mice with pTrMA (1 mg/kg i.v.) or lipopolysaccharides (LPS) (1 mg/kg i.p.), a positive control to cause inflammation, and repeating the ^18^F-FDG i.v. injection 24 h later. While LPS did cause inflammation that resulted in 2–4-fold increases in signal intensity for the liver, intestine, spleen, and bone marrow, there was no significant change in FDG signal compared to the baseline in any organ with measurable signal from the mice injected with pTrMA (Figures S3–S5).

**Figure 5:**
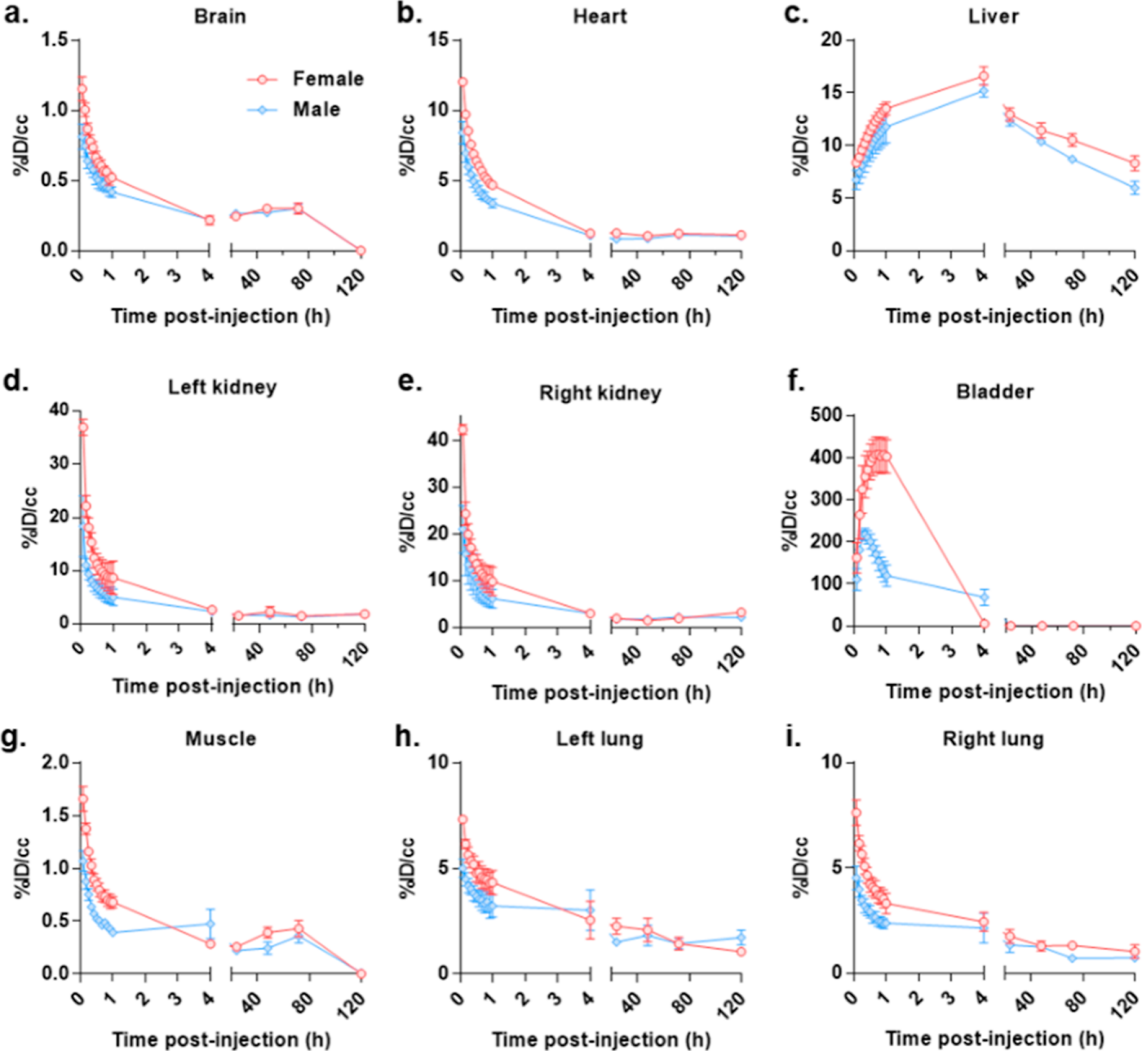
Time course biodistribution and excretion of ^64^Cu-labeled pTrMA-*co*-DOTA (200 μCi) from the organs (a) brain, (b) heart, (c) liver, (d) left kidney, (e) right kidney, (f) bladder, (g) muscle, (h) left lung, and (i) right lung of female (red, *n* = 4) and male (blue, *n* = 4) mice. Data were quantified from μPET images.

The time course biodistribution and excretion of the trehalose polymer was also studied in vivo with micropositron emission tomography (μPET) co-registered with microcomputer tomography (μCT) images for the anatomical information and attenuation correction. Mice (female *n* = 4 and male *n* = 4) were injected intravenously (i.v.) with the ^64^Cu-labeled pTrMA-*co*-DOTA (200 μCi) and underwent a series of μPET/μCT scans beginning with a 1 h dynamic scan, followed by static scans at 4, 24, 48, 72, and 120 h post-injection (Figures 4b,c and S6–S13 for all the mice and time points). Radioactivity measured as a percent of injected dose per cubic centimeter (% ID/cc) showed that pTrMA-*co*-DOTA was 77.5 ± 0.4 and 79.6 ± 2.0% excreted from female and male mice after 24 h, respectively. Dynamic scans over the first hour clearly show renal clearance of the majority of the ^64^Cu signal from labeled pTrMA-*co*-DOTA. The signal from the radiolabeled material moved through the kidneys, accumulated temporarily in the bladder, before being excreted from the animal. pTrMA-*co*-DOTA was distributed symmetrically in paired organs. The final μPET and μCT time point of the mice showed minimal signals of 8.3 ± 1.4 and 6.0 ± 1.2% in the liver and 3.0 ± 0.4 and 2.0 ± 0.3% in the kidneys of female and male mice, respectively, indicating incomplete clearance of the polymer with some accumulation in the liver for the duration of the study (Figure 5).

**Figure 6:**
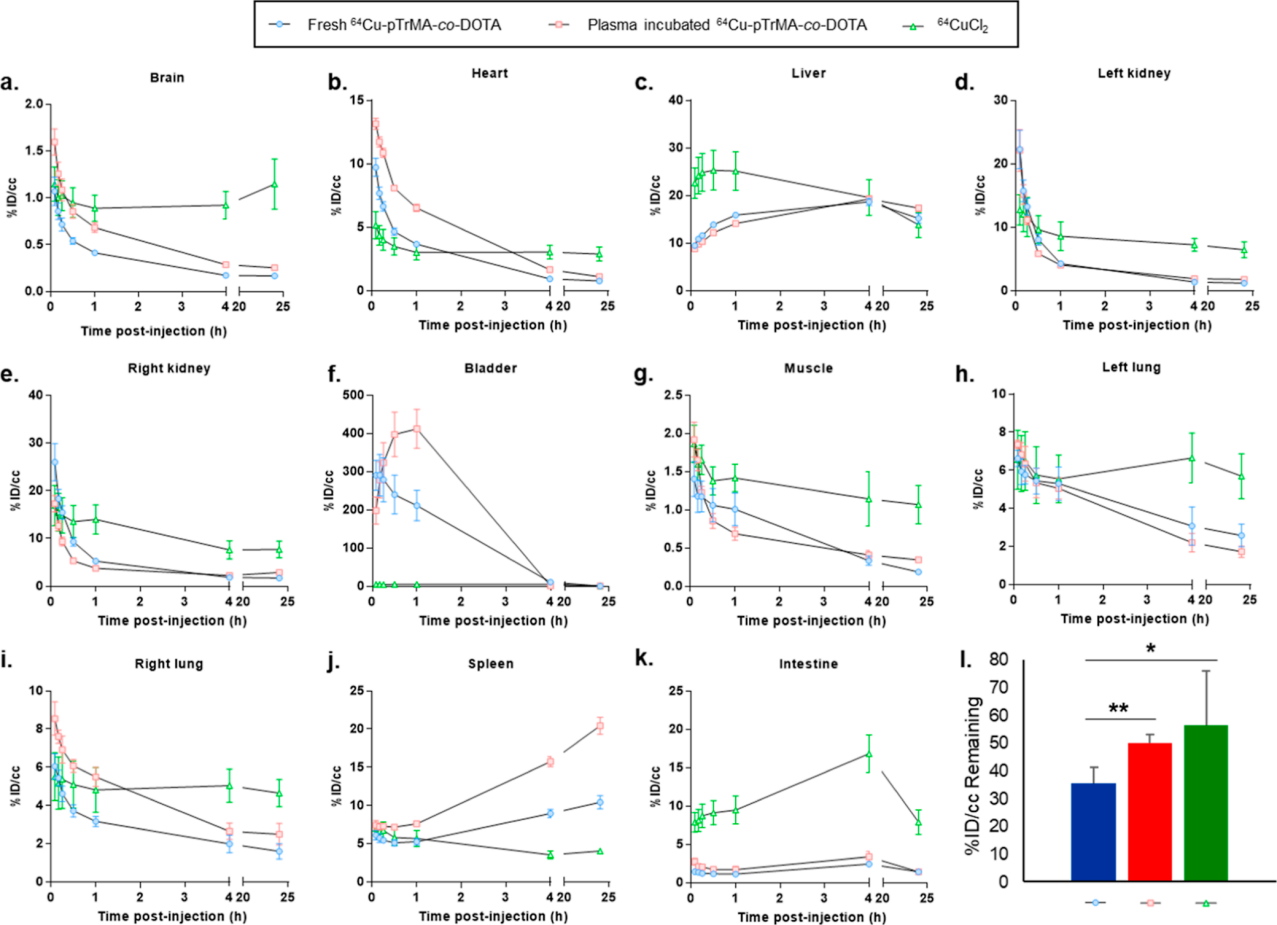
Time course biodistribution and excretion of fresh ^64^Cu-labeled pTrMA-*co*-DOTA (blue), plasma-incubated ^64^Cu-labeled pTrMA-*co*-DOTA (red), and free ^64^CuCl_2_ (green) from the organs (a) brain, (b) heart, (c) liver, (d) left kidney, (e) right kidney, (f) bladder, (g) muscle, (h) left lung, (i) right lung, (j) spleen, and (k) intestine of female (*n* = 4) mice. (l) Total signal remaining for each condition after 24 h (*p < 0.05 and **p < 0.01, with horizontal lines indicating the significantly different conditions). Data were quantified from μPET images.

To determine if organ signal attenuation was occurring because ^64^Cu was released from pTrMA-co-DOTA in vivo due to insufficiently strong DOTA chelation, the stability of the radiolabeled polymer as well as the biodistribution and excretion of free ^64^Cu was investigated by further μPET/μCT imaging. Mice (female, *n* = 4) were i.v. injected with the fresh ^64^Cu-labeled pTrMA-*co*-DOTA (200 μCi), ^64^Cu-pTrMA-*co*-DOTA after incubating in plasma at 37 °C for 24 h (100 μCi), and free ^64^CuCl_2_ (100 μCi). Post-injection, the mice were immediately scanned for 1 h with dynamic μPET, followed by a μCT scan; subsequently, they were scanned statically at 4 h (10 min) and 24 h (20 min) (Figure 6 and Figures S14–S27 for the same figures larger for better viewing). We are able to conclude that ^64^Cu-pTrMA-*co*-DOTA is largely stable in the plasma, as the biodistribution patterns between the two groups exposed to the ^64^Cu-pTrMA-*co*-DOTA is similar especially when compared to ^64^CuCl_2_. If ^64^Cu was readily leached from the DOTA group when incubated in plasma, the shifting biodistribution in the organs with time would have been the same as with the mice exposed to free ^64^CuCl_2_. However, the intensity from the 24 h time point showed that, while 35.6 ± 5.7% of ^64^Cu-pTrMA-*co*-DOTA remained, 50.3 ± 2.9 and 56.3 ± 19.7% signal remained from the plasma-incubated ^64^Cu-pTrMA-*co*-DOTA and ^64^CuCl_2_, respectively (Figure 6l). Additionally, because free ^64^Cu has high liver accumulation, the lack of a significant difference in liver accumulation between fresh and plasma-incubated ^64^Cu-pTrMA-*co*-DOTA indicates that its attenuated liver signal is not due to the released ^64^Cu. Additionally, there is an interesting accumulation of signal in the spleen from mice injected with the plasma-incubated ^64^Cu-pTrMA-co-DOTA that is not apparent in the other groups. While immunoglobulins are known to accumulate in the spleen, given the immunogenicity results and that the polymer was incubated ex vivo in naïve serum, attributing this accumulation to immunoglobulins binding would only be valid in the unlikely case of non-specific binding.^34^ While this result was curious, further exploration did not seem warranted as plasma incubation would not be a normal part of pTrMA use.

### Insulin Plasma Lifetimes with pTrMA

**Figure 7:**
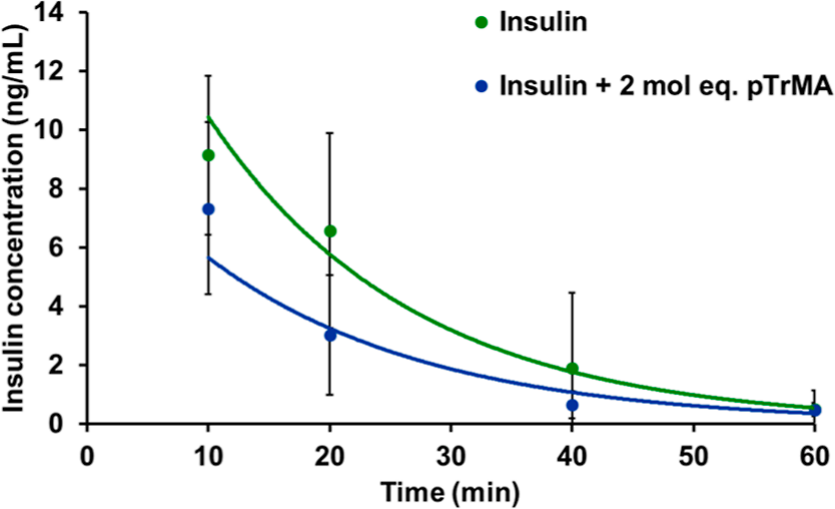
Plasma lifetime of insulin (120 μg/kg) injected i.v. in mice (*n* = 4) alone and with 2 mol equiv of pTrMA measured by insulin ELISA. No significant difference between the two conditions was detected at any time points.

Excipients should not change the pharmacokinetics of biologics. To investigate this, insulin levels in vivo (*n* = 4) over time were determined by ELISA for mice injected with insulin alone or insulin with 2 mol equiv of pTrMA. The plasma lifetime of insulin did not change from the addition of the excipient pTrMA at any time point (Figure 7), suggesting that the pharmacokinetics of insulin does not change with the addition of pTrMA.

### Mechanism of Insulin Stabilization to Simulated Accelerated Storage and Standard Storage Conditions with pTrMA

**Figure 8:**
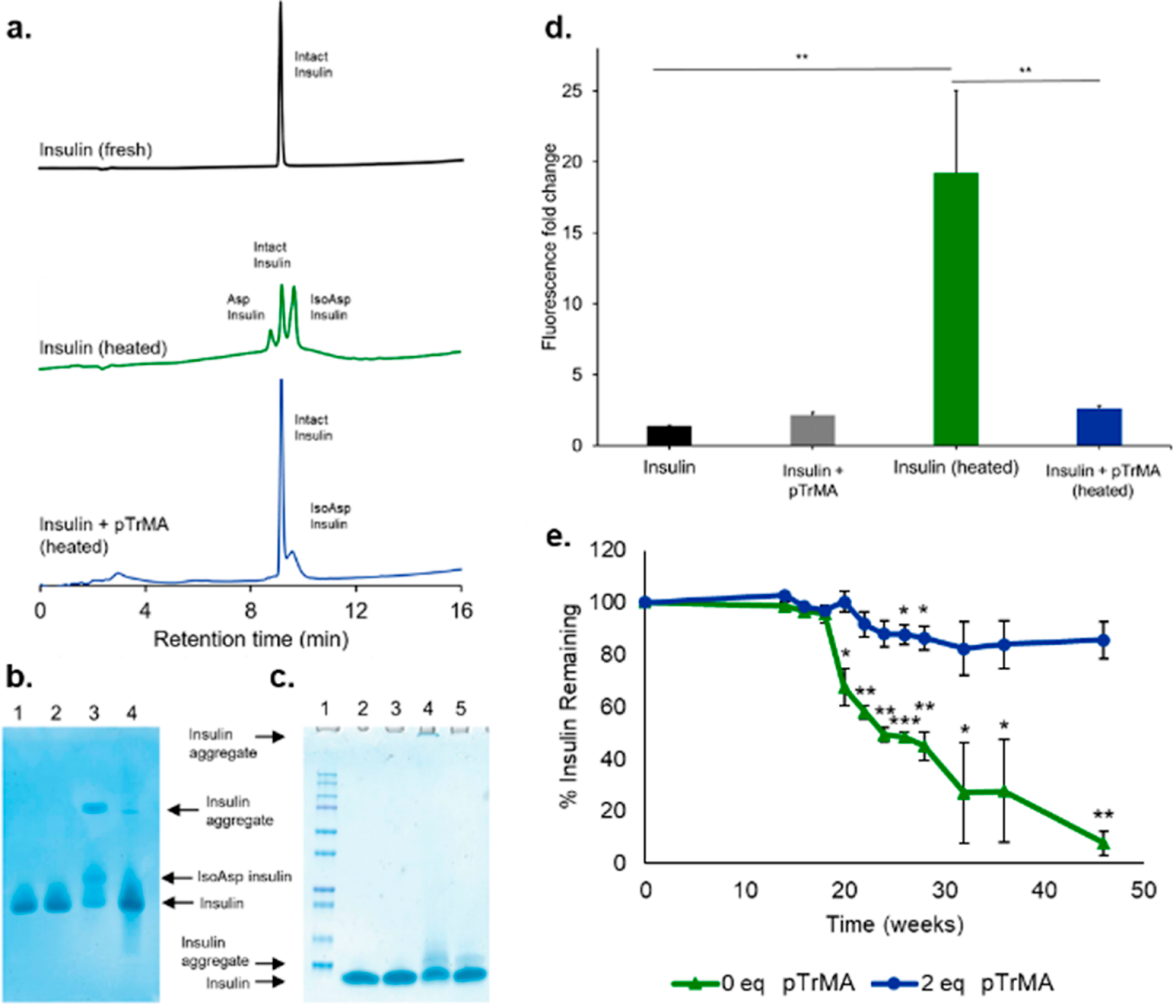
Characterization of insulin alone or in the presence of 2 mol equiv of pTrMA after exposure to heat (90 °C) for 30 min by (a) HPLC, (b) native PAGE gel [(1) insulin (fresh), (2) insulin + 2 mol equiv of pTrMA (fresh), (3) insulin (heated), and (4) insulin + 2 mol equiv of pTrMA (heated)], (c) SDS-PAGE [(1) protein dual-color ladder (10, 15, 20, 25, 37, 50, 75, 100, 150, and 250 kDa standard bands), 2–5 same as (b) native PAGE gel], and (d) ThT assay (*n* = 3). (e) Quantification of the remaining intact insulin alone or in the presence of 2 mol equiv of pTrMA (*n* = 3) after mild storage conditions (4 °C) for 46 weeks by HPLC (difference compared to the original time point). For all *p < 0.05, **p < 0.01, and ***p < 0.001.

Previous stabilization studies have found that the trehalose polymers stabilize insulin to heat and heat plus mechanical agitation stress conditions.^26−31^ To elucidate the mechanism, a standard accelerated heating assay was applied to insulin and insulin with 2 mol equiv of pTrMA. The formulations were heated to 90 °C for 30 min. The samples were then analyzed for degradation specifically by deamidation with HPLC using conditions that separate degraded insulin from the intact species.^5^ After heating, insulin alone appeared to degrade extensively at what has been identified as the AsnB3 site, resulting in Asp and isoAsp insulin derivatives with very little intact insulin remaining (Figure 8a). The added pTrMA prevented most degradation at AsnB3 with no Asp insulin present and only minimal isoAsp insulin formation. The heated samples were also investigated by native polyacrylate gel electrophoresis (PAGE) for changes to MW or isoelectric point for evidence of chemical changes or aggregate formation (Figure 8b). PAGE gel confirmed the HPLC analysis that both insulin chemical degradation products form with heat and that 2 mol equiv of pTrMA decreases the degradation. Additionally, native PAGE gel indicated that aggregates had formed as observed by the higher MW species in the gel, as did SDS-PAGE (Figure 8c). However, insulin heated alone resulted in the formation of both Asp and isoAsp insulin derivatives as well as insulin aggregates.

Fibrillation and aggregation of insulin were investigated by the thioflavin T (ThT) assay, where the ThT molecule binds to the available amyloids or misfolded proteins causing a shift in its emission spectrum to strongly fluoresce. Heated insulin produced significantly higher (*p* < 0.01) fluorescence signal than fresh insulin or insulin heated with pTrMA indicative of the unfolded insulin forming fibrils that the ThT could bind (Figure 8d). Notably, the insulin heated with pTrMA was not significantly different from the fresh insulin with pTrMA, although both pTrMA solutions were higher than fresh insulin alone (fresh *p* < 0.05 and heated *p* < 0.01). SEC-MALS analysis of insulin alone and with pTrMA was performed to investigate the oligomerization state of insulin.^35,36^ Monomeric and dimeric insulin were not resolved by SEC and co-eluted in an overlapping peak (Figure S28). However, no hexameric insulin was present with insulin alone or when pTrMA was added, and, interestingly, the remaining soluble insulin after heating was still monomeric or dimeric in apparently the same relative concentrations for insulin with and without pTrMA (all aggregated insulin was filtered out). In addition, differential scanning calorimetry was undertaken to study the melting temperature of the insulin. It was found that both pTrMA and trehalose shifted the melting temperature very slightly and to a similar extent compared to insulin (88.3 and 88.8 vs 86.6 °C, respectively, Figure S29). Thus, it is unlikely that the change in melting temperature is a significant factor in protein stabilization.

An extended storage assay was applied to insulin alone and insulin with 2 mol equiv of pTrMA to measure and correlate the effects of realistic storage conditions for comparison with accelerated aging. To study relevant storage, the formulations were kept at a standard refrigeration temperature at 4 °C for 46 weeks. After 14 weeks, the samples were analyzed for the intact insulin with HPLC conditions to separate out the degraded insulin (Figure 8e).^5^ After 20 weeks, insulin incubated alone significantly degraded to 67.4 ± 7.0% of the original level (*p* = 0.023 compared to the original time point), while insulin with pTrMA did not significantly differ from the original time point (100.3 ± 4.0%). Over the subsequent 26 weeks, as the percent intact insulin alone dropped, the solutions became visually cloudy and noticeably more difficult to filter before HPLC indicating aggregation. At the final time point (week 46), only 7.8 ± 4.6% of the insulin alone was intact. However, the level of insulin formulated with pTrMA only decreased by 13.0 ± 7.1% over the whole 46 weeks at 4 °C.

## Discussion

pTrMA is shown in these studies to be a safe excipient for stabilizing insulin. While the immunogenic protein OVA elicited a clear IgG and IgM antibody response alone and in the presence of pTrMA, no significant immune response to the polymer was found for the polymer alone or with OVA. Also, no significant cytokine response to pTrMA was detected. The expanded inflammation study by ^18^F-FDG corroborated the ELISA-based immunogenicity results by showing that pTrMA did not cause inflammation, and it does not appear to localize in inflamed organs. Based on these observations, pTrMA does not trigger antibody production as a host immune response or elicit increased inflammatory cytokine production. This is to be expected as both components are generally benign—trehalose is Generally Regarded As Safe (GRAS) by the FDA and polymethacrylate backbones have been utilized in human medicine since before the 1950s with the introduction of PMMA contact lenses.^37^ While PMMA contacts were replaced with softer polymer materials, PMMA is still commonly used in dentistry and orthopedics as bone cement, fillers, and vertebrae stabilization because of its biocompatibility and advantageous versatility from both physical and chemical perspectives. More recent iterations of PMMA bone cement have even been used to deliver osteogenic growth factors.^38^

A total of 77.5 ± 0.4 and 79.6 ± 2.0% of the radioactivity was cleared primarily via renal excretion within 24 h from female and male mice, respectively. ^64^Cu has been shown to stably accumulate in the liver (13% ID/cc) of C57HL/6 mice with concurrent accumulation in the kidneys (10% ID/cc) at 72 h.^39^ μPET/μCT of the Cu^64^-radiolabeled pTrMA revealed some signal attenuation in the liver even at the final time point, 8.3 ± 1.4 and 6.0 ± 1.2%, respectively. In the final time points of the extended μPET/μCT experiment, the signal in the liver still appeared to be going down, while the study could not be extended due to the limited radioactive half-life, it is possible that pTrMA was still being excreted. ^14^C labeling of the polymer may be necessary to fully understand the excretion and metabolism of the polymer in future studies.^40^ These results are similar to the results with DOTA-conjugated PEG-based star and linear polymers from the Wooley, Hawker, and Welch labs with some accumulation in the liver and kidneys across a range of polymer chemistries and architectural designs.^41−45^ Like our 10 kDa linear copolymer, nanoparticles formed from poly(methyl methacrylate) and poly(ethylene glycol) comb copolymers (1–5 kDa) showed attenuation in the liver, spleen, and kidneys even at 50 h.^42^ Alone, our data could not reveal finally whether the DOTA chelating ligand was leaching ^64^Cu in vivo, but additional experiments by μPET/μCT could be used to explore this possibility in the future, including using an alternative metal and chelators. Finally, the addition of trehalose polymer as an excipient did not alter the in vivo plasma lifetime of insulin, indicating that it would not cause a delay in bioactivity. The lack of anti-pTrMA IgG or IgM antibodies combined with the majority clearance of pTrMA and no significant impact on insulin in vivo lifetime indicate that the polymer is safe to use as an excipient and that extensive toxicity studies and further safety studies including metabolism are warranted.

While in vivo safety is critical, the reason to employ pTrMA is to provide stabilization to the therapeutic protein or peptide. Insulin was stabilized against heat-induced chemical degradation and aggregation by pTrMA according to HPLC, ThT assay, and native PAGE gel. All the three assays indicated that the trehalose polymer decreased the aggregation of insulin. Furthermore, HPLC and native PAGE gel analysis demonstrated that the deamidation of insulin was inhibited, and PAGE gel indicated intact insulin to be present. Fibrillation of insulin is accelerated when the conformational stability is decreased, such as through exposure to heat, low pH, or the air–water interface during mechanical agitation.^5,7^ It should be noted that in all the stability studies, the protein was not protected from ambient light. It is known that insulin stability is affected by UV light exposure, and in future studies, this will need to be studied separately as a stressor.^46,47^ SEC-MALS of fresh insulin indicated that the addition of pTrMA did not shift the monomeric and dimeric insulin into the more complexed hexameric assembly, and the relative concentrations did not appear to be affected by exposure to heat stress. These results indicate that pTrMA is maintaining the stability of monomeric and dimeric insulin, the less stable conformations. By stabilizing insulin without complexing into the hexameric state, pTrMA would most likely not increase delay in the onset of insulin activity due to the time required for insulin to disassemble from greater oligomeric states into the active monomeric state, and this will need to be tested in future studies. Not indicating a likelihood for delayed onset of activity is important in particular with diabetic patients because delays between dosing and onset of bioactivity can result in hyperglycemic incidents that can harm tissues.

While insulin complexation with pTrMA was not detected by SEC-MALS (or in vivo plasma lifetime) in this study, it is unknown at this time how pTrMA stabilizes insulin without strongly interacting with it. However, SEC-MALS is limited in resolution and any aggregate is removed by filtration prior to the study. Better understanding would necessitate studies such as nuclear magnetic resonance (NMR) spectroscopy analysis to identify intermolecular interactions by insulin chemical shift perturbations. While the effect of polymer chain lengths as well as polymer concentrations has been shown to increase stabilization with both concentration and chain length, further exploration could elucidate the importance of polyvalence-type stabilization for the trehalose polymer. DSC did not indicate a major shift in insulin melting temperature in the presence of the polymer. Further analysis should include thermal shift by fluorimetry to track protein unfolding when stabilized by the trehalose polymer. Other forms of particle characterization and sizing (including dynamic light scattering and micro-flow imaging) could also be used to further understand how pTrMA prevents insulin from undergoing the aggregation and fibrillation pathway that leads to loss in activity. In a previous work using horseradish peroxidase, we reported that a similar methacrylate trehalose polymer made with an ether linkage acted as a chaperone and helped a protein refold after heating.^22^ It is possible that pTrMA may improve the conformational stability of insulin, and of insulin monomer in particular, to prevent aggregation by a similar mechanism or by preventing air–solution or air–glass destabilizing, interfacial interactions. Inhibition of heat-induced deamidation by pTrMA may also be related to conformational stability of the protein, as solvent accessibility/flexibility around relevant residues is related to the rate of deamidation in proteins.^48^ What we do know is that the polymer does prevent chemical change and aggregation in insulin and does stabilize the protein to a variety of stressors including heat, mechanical agitation, and long-term refrigeration storage.

Overall, the results herein demonstrate that pTrMA-formulated insulin is promising especially for use in insulin pumps where formulations are exposed to body and elevated temperatures for prolonged periods at the skin surface, and strenuous activity can cause mechanical agitation for the insulin within the pump reservoir. Further, the safety and stabilization capabilities of pTrMA as an excipient with insulin suggest that it could be expanded to other therapeutic biologics, making it promising for formulation application in the pharmaceutical industry.

## Conclusions

As insulin continues to be major therapeutic for treating diabetes, and diabetes is increasing worldwide, the need for new stabilizing excipients for the peptide continues to increase. Insulin needs to not only be stabilized against environmental stressors but also be available in its active, less-stable, monomeric state to reduce delays in the onset of activity post-administration. Biodistribution of the trehalose-based glycopolymer pTrMA by PET and immunogenicity by ELISA and multiplexed ELISA indicated that pTrMA resides only minimally in the liver, similarly to other polymers, and does not cause immunogenic clearance responses (innate or adaptive). Insulin injected with pTrMA had the same plasma lifetime profile as free insulin, further indicating that pTrMA does not affect in vivo properties. Stability studies into the mechanism by which insulin is stabilized demonstrated inhibition of the known chemical degradation pathways, as well as prevention of aggregation pathways. Investigation of the oligomeric state of insulin showed that pTrMA did not shift the insulin to the slower onset of activity hexameric state even after heat stress. Long-term stability studies at 4 °C demonstrated that with just pTrMA, insulin was stabilized for at least 46 weeks at pH 7.4. This work clearly illustrates that pTrMA should be further explored for use in the clinic as an insulin excipient.

## Experimental Section

### Materials

All chemicals purchased from Sigma-Aldrich or Fisher Scientific were used without further purification unless mentioned otherwise. Anhydrous compounds were dried over molecular sieves. Azobis(isobutyronitrile) (AIBN) was recrystallized from acetone before use. Trehalose purchased from BulkSupplements.com (Henderson, NV) was repeatedly dried azeotropically from ethanol and stored under vacuum. Spectra/Por3 regenerated cellulose membrane (MWCO 3.5 or 1.0 kDa) used for polymer dialysis was purchased from Spectrum Chemical (New Brunswick, NJ). (2,2′,2″-(10-(2-((2-(2,5-Dioxo-2,5-dihydro-1*H*-pyrrol-1-yl)ethyl)amino)-2-oxoethyl)-1,4,7,10-tetraazacyclododecane-1,4,7-triyl)triacetic acid) (maleimide-DOTA) was purchased from CheMatech and used as received. Sterile, endotoxin-free, chicken egg white OVA containing <1 endotoxin unit (EU)/mg was purchased from InvivoGen (EndoFit, Version 17E10-MM, EFP-41-02, 98% purity minimum) and reconstituted with EU-free, sterile, saline solution according to the manufacturer’s protocol. Recombinant human insulin was purchased from Sigma-Aldrich (lot no. 17N331). BSA, heat-shock-treated, was purchased from Fisher Scientific (lot no. 43233). CupriSorb was purchased from Seachem. Goat anti-mouse IgG HRP conjugate and goat anti-mouse IgM HRP conjugate were both purchased from Abcam, reconstituted, diluted 2× with glycerol, and stored at −80 °C following manufacturer’s recommendations. Pierce BCA assay kit and enzyme-linked immunosorbent assay (ELISA) TMB development solution were purchased from Thermo Fisher Scientific. Insulin ELISA kit was purchased from Mercodia. The kD Mini-PROTEAN-TGX PAGE gels and the SDS-PAGE protein standards (Precision Plus ProteinTM Dual Color) were purchased from Bio-Rad. Amicon Centriprep tubes were purchased from Millipore. ^64^Cu radioisotope was purchased from Washington University School of Medicine. Initiators, methacrylate trehalose monomer (TrMA), and polymers except pTrMA-*co*-DOTA were synthesized as previously described, and their synthesis is briefly described below.^22,28,49^

### Analytical Techniques

NMR spectroscopy was performed on a Bruker AV 400 MHz, DRX 500 MHz, or AV 500 MHz instrument. ^1^H NMR spectra were acquired with a relaxation delay of 2 s for small molecules and 30 s for polymers. ^13^C NMR spectra were acquired with a relaxation delay of 30 s for polymers. Preparatory reversed-phase HPLC purification was performed on a Shimadzu HPLC system equipped with a UV detector using a Luna 5 μm C18 100A column (preparatory: 5 μm, 250 × 21.2 mm) with monitoring at λ = 215 nm and 254 nm. Aqueous gel permeation chromatography (GPC) was conducted on a Malvern Viscotek GPCMax system equipped with a triple detector array (TDA 305-040 Quadruple Detector Array), with two Viscotek A600 M general mixed aq. columns (300 × 8.0 mm). The samples were injected at 5 mg/mL and run with water and 20% methanol (MeOH) at a flow rate of 1 mL/min. The RI detector used a d*n*/d*c* value of 0.15, and calibration was performed with near-monodisperse PEG standards from Polymer Labs. Fast protein liquid chromatography (FPLC) to purify BSA conjugates was performed on a Bio-Rad BioLogic DuoFlow chromatography system equipped with a HiTrap Q HP (1 mL) cation-exchange column from GE Healthcare with an eluent of 20 mM Tris buffer pH 8.6 from 0 to 1 M NaCl. Immune ELISA assay results were read on an ELX800 Universal Microplate Reader (Bio-Tek Instrument Inc., Winooski) with λ = 450 and 630 nm for signal and background, respectively. μPET imaging was performed with a Siemens Inveon μPET scanner, and the corresponding μCT was performed on a CrumpCAT scanner (UCLA Crump Institute for Molecular Imaging). Analytical HPLC for insulin detection was conducted on an Agilent 1260 Infinity II LC System equipped with a UV detector and a Zorbax 300 SB-C3 column with a gradient of 0–100% acetonitrile in water + 0.1% trifluoroacetic acid over 17 min unless otherwise noted. ThT fluorescence assay results were read on a Tecan Infinite M1000 Pro equipped with a Tecan Quad4 Monochromator. Size exclusion chromatography coupled with multi-angle light scattering (SEC-MALS) was performed on insulin samples with a General Electric Healthcare ÄKTA Pure and Wyatt Technology miniDawn TREOS in tandem with an Optilab T-rEX refractometer with extended range. SEC-MALS was equipped with a Wyatt WTC-030S5 SN 0830 SEC column with an eluent of 0.25 M PBS pH 7.4. DSC was conducted on a Mettler Toledo DSC+ equipped with a HSS 9+ Ceramic Sensor.

### Animal Usage

All animal experiments were conducted according to the protocols approved by the UCLA Animal Research Committee (ARC).

### Methods

#### Synthesis of TrMA

The methacrylate-functionalized trehalose monomer was synthesized according to the literature procedure.^22^ In a flame-dried round-bottom flask, trehalose (4.6 g, 13.4 mmol) was dissolved in anhydrous dimethyl sulfoxide (DMSO, 60 mL, purity ≥ 99%). Anhydrous triethylamine (TEA, 5.6 mL, 40.2 mmol, purity ≥ 99%) and methacrylic anhydride (400 μL, 2.7 mmol, purity 94%) were added, and the mixture was stirred at 23 °C for 18 h. DMSO was removed by precipitating into 0 °C hexanes and dichloromethane (hexanes/DCM, 8:2, 1400 mL). The organic layer was decanted, and the remaining organics were removed from the viscous liquid by rotary evaporation. The crude product was diluted with water before filtering and purifying by preparatory HPLC with a gradient of MeOH in water (10–50%). To the product containing fractions, mequinol was added to prevent autopolymerization. The solvent was removed by rotary evaporation using a two-neck flask equipped with a septa and a long needle directly into the solution to further prevent autopolymerization by providing a source of oxygen. The product was finally recovered by lyophilization (460.6 mg, 42% yield). ^1^H NMR (400 MHz in D_2_O): δ 6.03 (s, 1H), 5.62, (s, 1H), 5.07 (d, *J* = 3.9 Hz, 1H), 5.03 (d, *J* = 3.9 Hz, 1H), 4.38 (dd, *J* = 12.2, 2.2 Hz, 1H), 4.24 (dd, *J* = 12.2, 5.2 Hz, 1H), 3.96 (qd, *J* = 10.2, 1.8 Hz, 1H), 3.71 (m, 4H), 3.63 (m, 1H), 3.52 (ddd, *J* = 19.6, 10.2, 3.9 Hz, 1H), 3.42 (dd, *J* = 10.2, 9.3 Hz, 1H), 3.32 (t, *J* = 9.3 Hz, 1H), 1.82 (s, 3H). ^1^H NMR spectrum agreed with the one previously reported (Figure S30).

#### Synthesis of 2-Hydroxyethyl 2-Bromoisobutyrate

2-Hydroxyethyl 2-bromoisobutyrate (HEBIB) was synthesized according to the literature procedure.^50^ Ethylene glycol (1.33 mL, 23.8 mmol, anhydrous, purity 99.8%) and anhydrous TEA (0.66 mL, 4.74 mmol, purity ≥ 99%) were stirred together and cooled to 0 °C before dropwise addition of α-bromoisobutyryl bromide (0.5 mL, 4.8 mmol, purity 98%). The reaction mixture was stirred for 2 h at 0 °C before warming up to 23 °C and stirred for an additional 12 h. Water (10 mL) was added to the flask and extracted with chloroform three times (10 mL each time). The chloroform layer was washed with dilute hydrochloric acid, saturated sodium bicarbonate (NaHCO_3_), and water (10 mL each wash). The organic layer was dried over anhydrous magnesium sulfate (MgSO_4_) and filtered, and the product was recovered after rotary evaporation. The product was purified by silica gel flash chromatography with an eluent system of diethyl ether and hexanes (1:1). The product-containing fractions were collected and recovered the product as a yellow clear oil by rotary evaporation (658 mg, 65% yield). ^1^H NMR (400 MHz in CDCl_3_): δ 4.32 (t, *J* = 4.6 Hz, 2H), 3.87 (t, *J* = 4.6 Hz, 2H), 1.95 (s, 6H), 1.86 (s, 1H). ^1^H NMR spectrum agreed with that reported for this compound (Figure S31).

#### Synthesis of (4-Formylphenyl-2-bromoisobutyrylate) Initiator

(4-Formylphenyl-2-bromoisobutyrylate) (benzaldehyde initiator) was synthesized according to the literature procedure.^51^ 4-Hydroxybenzaldehyde (174 mg, 1.4 mmol, purity 98%) was dissolved in anhydrous dimethylformamide (4 mL, purity 99.8%) under argon. Anhydrous TEA (0.2 mL, 1.4 mmol, purity ≥ 99%) was added and stirred at 0 °C for 10 min before dropwise addition of α-bromoisobutyryl bromide (0.1 mL, 1.4 mmol, purity 98%). The reaction mixture was stirred for 30 min at 0 °C before warming up to 23 °C and stirred for an additional 14 h. The white precipitate that formed was filtered off, and the remaining filtrate was concentrated by rotary evaporation. The crude material was dissolved in DCM and washed twice with saturated NaHCO_3_. The organic layer was dried over anhydrous MgSO_4_ and filtered, and the product was recovered after rotary evaporation. The product was purified by silica gel flash chromatography with an eluent system of diethyl ether and ethyl acetate (1:4). The product-containing fractions were collected and recovered by rotary evaporation (86 mg, 33% yield). ^1^H NMR (400 MHz in CDCl_3_): δ 10.03 (s, 1H), 7.96 (t, *J* = 4.6 Hz, 2H), 7.35 (t, *J* = 4.6 Hz, 2H), 2.08 (s, 6H). ^1^H NMR spectrum agreed with that reported for this compound (Figure S32).

#### AGET ATRP of TrMA with HEBIB Initiator for Use as an Excipient

Polymerization was performed according to the literature procedure.^28^ Dulbecco’s PBS (DPBS, 0.1 M) pH 7.4 was degassed by sparging with argon for at least 1 h. Ascorbic acid (AA) was dissolved in DPBS (10 mg/mL) and degassed for >30 min. TrMA (50 mg, 120 μmol) was added directly to a Schlenk flask equipped with a stir bar, and the flask was evacuated and refilled with argon four times. Stock solutions of copper(II) bromide (CuBr_2_, purity 99%) and tris(2-pyridylmethyl)amine (TPMA, purity 98%) were prepared using degassed DPBS at 5 and 6.75 mg/mL, respectively. The HEBIB initiator (0.23 mg, 1.1 μmol) was dissolved in a requisite amount of each of the CuBr_2_ and TPMA solutions (0.24 mg, 1.1 μmol and 0.31 mg, 1.1 μmol) and then transferred to the flask under argon. AA solution (0.1 mg, 0.6 μmol) was added to the flask under argon to initiate the polymerization, with a final TrMA concentration of 0.45 M. The polymerization proceeded under argon at 25 °C for 7.5 h. The polymerization was ended by exposing to air, and the polymer was dialyzed against water (3.5 kDa MWCO) with CupriSorb, a metal-chelating resin, to chelate copper for 2 days (8 L of water). The polymer was recovered as a fluffy white solid by lyophilization (27.1 mg, 65% yield). ^1^H NMR (400 MHz in D_2_O): δ 5.10, 5.07, 4.24, 4.02, 3.94, 3.75, 3.53, 3.35, 1.83, 1.51, 0.96, 0.80. Number-average MW (*M*_n_) = 10.1 kDa (by GPC with PEG standards), MW dispersity (*Đ*) = 1.25. ^1^H NMR spectrum agreed with that previously reported (Figure S33). The GPC spectrum is shown in Figure S34.

#### AGET ATRP of TrMA with Benzaldehyde Initiator for Conjugation to BSA

Polymerization was performed according to the literature procedure.^28^ Water and MeOH (1:1) were degassed by sparging with argon for 40 min. TrMA (150 mg, 0.37 mmol) was added directly to a Schlenk flask equipped with a stir bar, and the flask was evacuated and refilled with argon four times. CuBr_2_ (purity 99%) and TPMA (purity 98%) were dissolved in degassed water and MeOH as 34.0 and 44.2 mg/mL, respectively. The benzaldehyde initiator (4.1 mg, 15 μmol) was dissolved in CuBr_2_/TPMA solution (3.4 mg, 15 μmol and 4.42 mg, 15 μmol) and then transferred to the flask under argon. AA was dissolved in degassed water and MeOH solution (3 mg/mL), and an aliquot of this solution (1.6 mg, 9 μmol) was transferred to the flask under argon to initiate the polymerization, with a final concentration of 0.45 M TrMA. The polymerization proceeded under argon at 25 °C for 15 h. The polymerization was ended by exposing to air, and the polymer was dialyzed against water and MeOH (1:1, 3.5 kDa MWCO) for 2 days (8 L of water, 1 day with CupriSorb). The polymer was recovered via lyophilization (138.1 mg, 92% yield). ^1^H NMR (400 MHz in D_2_O): δ 9.86, 7.96, 7.26, 5.10, 5.06, 4.24, 4.01, 3.92, 3.75, 3.53, 3.35, 1.82, 1.53, 0.98, 0.80. *M*_n_ = 24.9 kDa (by GPC with PEG standards), *Đ* = 1.05. ^1^H NMR spectrum agreed with that previously reported (Figure S35). The GPC spectrum is shown in Figure S36.

### FRP Copolymerization of TrMA and Pyridyl Disulfide Rther Methacrylate for Studying Biodistribution and Excretion

Polymerization was performed according to the literature procedure.^49^ TrMA (75 mg, 0.18 mmol) and pyridyl disulfide ether methacrylate monomer (PDSMA, 1.9 mg, 7.5 μmol) were dissolved in a solution of AIBN in dry DMSO (10 mg/mL AIBN, 0.25 M TrMA). The solution was freeze–pump–thawed five times and polymerized at 80 °C for 20 h. The polymer was dialyzed in 3.5 kDa MWCO dialysis tubing against water containing CupriSorb for 2 days (8 L). Lyophilization yielded a random pTrMA-co-PDMSA copolymer with 0.85 PDSMA units per polymer chain as determined by combined ^1^H NMR and GPC analysis (81.3 mg, 100% yield). ^1^H NMR (500 MHz in D_2_O): δ 8.37, 7.82, 7.27, 5.10, 5.06, 4.24, 3.95, 3.76, 3.53, 3.36, 1.85, 1.55, 0.97, 0.81. ^13^C NMR (126 MHz in D_2_O): δ 93.4, 72.7, 72.1, 71.1, 69.7, 64.4, 60.7, 44.9, 38.7. *M*_n_ = 8.9 kDa (by GPC with PEG standards), *Đ* = 2.19 (Figures S37–S39).

#### Synthesis of pTrMA-*co*-DOTA via Post-polymerization Modification

Using metal-free water, a stock solution of (tris(2-carboxyethyl)phosphine) (TCEP, 12 mg/mL) was prepared. The polymer pTrMA-*co*-PDMSA (8.9 kDa, *Đ* = 2.19 by GPC, 20.3 mg, 2.3 μmol of polymer, 2.3 μmol of PDMSA) was dissolved in TCEP stock (3.3 mg, 11.4 μmol) and mixed at 25 °C and 350 rpm for 30 min. Small molecules were removed by Centriprep centrifugal filtration (3 kDa MWCO) three times with additional TCEP stock before diluting the polymer with TCEP solution. Maleimide–DOTA (10.0 mg, 18.9 μmol) was dissolved in metal-free Milli-Q and added to the reaction for a polymer concentration of 8.4 mM. The reaction was mixed in a ThermoShaker at 25 °C and 350 rpm for 36 h before dialyzing reaction in 3.5 kDa MWCO dialysis tubing against water containing CupriSorb (16 L, 30 h). The polymer was recovered via lyophilization (18.4 mg, 86% yield). ^1^H NMR (500 MHz in D_2_O): δ 5.11, 5.07, 4.24, 3.94, 3.75, 3.53, 3.35, 3.04, 2.91, 2.58, 2.48, 1.84, 0.96, 0.80. ^13^C NMR (126 MHz in D_2_O): δ 178.7, 177.8, 92.4, 71.8, 70.4, 68.9, 63.3, 59.9, 53.1, 51.6, 48.2, 44.0, 17.1, 15.5. *M*_n_ = 10.3 kDa (by GPC with PEG standards), *Đ* = 2.68 (Figures S40–S42).

#### Preparation of BSA Conjugate for IgG and IgM ELISA Assay

BSA (25 mg, 0.38 μmol) and benzaldehyde pTrMA (24.9 kDa, 55.2 mg, 2.2 μmol) were dissolved in 25 mM borate buffer, pH 8.5. This solution was incubated for 10 min at 23 °C on a rocker. Sodium cyanoborohydride (41.1 mg, 654.3 μmol) was added to the reaction, and this was mixed for 4 h at 37 °C with 300 rpm agitation. Small molecules were removed from the crude product by Centriprep centrifugal filtration (30 kDa MWCO) with 25 mM PBS buffer pH 7.4 four times. The conjugate was purified by cation-exchange FPLC, and product-containing fractions were pooled and buffer-exchanged to PBS pH 7.4 using Centriprep centrifugal filtration (MWCO 30 kDa). Some unconjugated BSA was still present along with a majority conjugate product, and the relative amount of unconjugated BSA was measured to be 4.9% using ImageJ (Figure S1). It was determined that the BSA conjugate could be used as received, given that the majority was the BSA conjugate and the subsequent step by ELISA is blocking the wells with unmodified BSA.

#### Determination of BSA Conjugate Concentration by BCA Assay

The BSA–pTrMA conjugate concentration was determined using the Pierce BCA assay kit according to manufacturer specifications. Briefly, the conjugate was diluted (10× and 100×), and 10 μL of sample and BSA standards (20–2000 μg/mL) were pipetted into a 96-well plate. The working reagent (200 μL, 50-parts A + 1-part B) was added to each well. The plate was incubated at 37 °C for 30 min and then cooled to room temperature before measuring the absorbance at 562 nm. The diluted BSA–pTrMA conjugate concentration was calculated from the standard curve (*R*^2^ = 0.9954) to be 18.4 mg/mL (final product: 10.0 mg, 40% yield).

#### Antibody Immunogenicity Study of OVA and pTrMA In Vivo

CD-1 mice (8 weeks, female, *n* = 5–6, Charles River Laboratories) were used to study the immunogenicity of pTrMA (10.1 kDa, *Đ* = 1.25) alone or with OVA, a known immunogenic protein. OVA antigen with or without pTrMA was administered via i.p. injection (2 mg/kg OVA, 10 wt % pTrMA in sterile saline buffer); pTrMA (10 wt % pTrMA in sterile saline buffer, 100 mg/mL) was also administered alone via i.p. injection. Mice were challenged again 2 weeks after inoculation with the same dosage for each condition. Blood was collected into serum separator centrifuge tubes (SSTs) via retro-orbital bleeding at 1, 2, and 3 weeks after administration. Blood was centrifuged at 2000 rcf for 15 min to extract the serum. The serum was stored at −80 °C until ELISA could be run. Mice were sacrificed after 4 weeks and a final time point was collected via cardiac puncture. Blood was treated the same as prior time points.

For the ELISA, sterile-filtered 0.1 M PBS buffer + 0.3% Tween-20 was used to wash the wells four times between each step, making sure to remove solution by hitting the plates against paper towels after each wash. Antigen solutions of OVA or the BSA–pTrMA conjugate (total 0.02 mg/mL, conjugate only 0.019 mg/mL, and 100 μL/well) were plated on 96-well plates. After incubating at 4 °C for 16 h, the antigen was removed, and the wells were washed and blocked with sterile-filtered 3% BSA in 0.1 M PBS (300 μL) for 2 h at 37 °C. After washing out the BSA blocking solution, the serum from the inoculated mice diluted 100, 500, 2500, and 12,500-fold with filtered 1% BSA in 0.1 M PBS (100 μL/well) was then incubated with the respective antigen for 2 h at 37 °C (OVA antigen with OVA-exposed mice, separately OVA antigen and the BSA–pTrMA conjugate with OVA/pTrMA-exposed mice, and BSA–pTrMA conjugate with pTrMA exposed mice). After washing the wells, goat anti-mouse IgG or IgM HRP-conjugate antibody diluted 2000× with filtered 1% BSA in 0.1 M PBS (100 μL/well) was incubated for 1 h at 25 °C. The secondary detection antibody HRP conjugate was removed, and the wells were washed. In the dark, TMB substrate solution was added (100 μL/well), and the plate was incubated at 23 °C. After 5–10 min, when the color had developed in positive control wells, the reaction was quenched by adding 2 M H_2_SO_4_ (50 μL/well) stop solution. Absorbance was measured at 450 nm and background at 570 nm on a Tecan plate reader. Controls included the OVA antigen with OVA-exposed mouse serum (positive control), OVA antigen with naïve mouse serum (negative control), BSA–pTrMA antigen with naïve mouse serum (negative control), and BSA antigen with naive mouse serum (negative control and check on non-specific BSA binding). Because multiple 96-well plates were used to capture the full range of data in these experiments, the plates were normalized based on the maximum and minimum absorbance in the positive and negative control wells, respectively.

#### Cytokine Immunogenicity of pTrMA

CD-1 mice (6 weeks, female, *n* = 5, Charles River Laboratories) were used to study the immunogenicity of pTrMA (33.8 kDa, *Đ* = 1.01, 10 mg/kg in DPBS) relative to the negative control DPBS administered via i.p. injection. Blood was collected into SSTs via retro-orbital bleeding 1 h and 6 h after administration. Blood was centrifuged at 2000 rcf for 10 min at 4 °C to extract serum. Mice were sacrificed after 24 h, and a final time point was collected via cardiac puncture. Blood was treated the same as prior time points. A multiplexed ELISA-type assay (Luminex xMAP) was employed to measure IL-1b, IL-2, IL-4, IL-6, KC, IL-10, IL-12, IFN-γ, and TNF-α at the UCLA Immune Assessment Core (Dept. Pathology and Laboratory Medicine directed by Dr. Maura Rossetti). It should be noted that two different trehalose polymers were investigated at the same time with the same PBS control. It was deemed not an ethical use of animals to repeat the experiments only to obtain new PBS controls. Therefore, the PBS controls shown in Figure 3 herein are the same as those in Figure 3 in ref (33).

#### Radiolabeling of pTrMA-*co*-DOTA

Dissolved pTrMA-*co*-DOTA (8.9 kDa) in 0.4 M ammonium acetate buffer pH 3.5 (1 mg/mL). Pipetted ^64^Cu (6 mCi) into a screw-cap polypropylene tube before adding pTrMA-*co*-DOTA solution (300 μL). The solution was mixed gently and set on heat at 50 °C for 30 min. The solution was diluted with PBS (3 mL) for a specific activity of 20 μCi/μg with a purity of >99%.

### μPET/μ-Computed Tomography (μCT) Extended the Study of ^64^Cu-labeled pTrMA-*co*-DOTA Biodistribution and Excretion

C57BL/6 mice (10 weeks, *n* = 4 female and *n* = 4 male, from UCLA Radiation Oncology Colony) were anesthetized with 1.5% vaporized isoflurane and injected with the ^64^Cu-labeled pTrMA-*co*-DOTA (200 μCi) via i.v. injection (tail vein), immediately followed by a 1 h dynamic μPET scan and a μCT scan. After the 1 h dynamic imaging, mice were then imaged by μPET/μCT at 4 h (static μPET, 10 min), 24 h (static μPET, 15 min), 48 h (static μPET, 20 min), 72 h (static μPET, 30 min), and 120 h (static μPET, 60 min) post-injection. All μPET images were acquired with an energy window of 350–650 keV, followed by three-dimensional (3D) histographic analysis and reconstruction using the 3D-ordered subset expectation maximization (OSEM)/maximum a posteriori (MAP) method. Data was decay-corrected and scaled by initial imaging. Co-registered μPET/μCT images were analyzed and maximum-intensity projections (MIPs) were generated using AMIDE software.

#### μPET/μCT Stability Study of ^64^Cu-Labeled pTrMA-*co*-DOTA for Biodistribution and Excretion

^64^Cu-radiolabeled pTrMA-*co*-DOTA was incubated in plasma collected from a female C57BL/6 mouse at 37 °C for 24 h. Three groups of C57BL/6 mice (8–12 weeks, female, *n* = 4, from UCLA Radiation Oncology Colony) were anesthetized with 1.5% vaporized isoflurane and injected via i.v. injection (tail vein) with ^64^Cu-labeled pTrMA-*co*-DOTA (200 μCi), ^64^Cu–CuCl_2_ (100 μCi), or plasma-incubated ^64^Cu-labeled pTrMA-*co*-DOTA (100 μCi). Each group was scanned immediately after injection for 1 h with a dynamic μPET, followed by a μCT scan. Mice were imaged by μPET/μCT again at 4 h (static μPET, 10 min) and 24 h (static μPET, 20 min). All μPET images were acquired with an energy window of 350–650 keV, followed by 3D histographic analysis and reconstruction using the 3D-OSEM/MAP method. Data was decay-corrected and scaled by initial imaging. Co-registered μPET/μCT images were analyzed, and MIPs were generated using AMIDE software.

#### μPET/μCT Study of pTrMA-*co*-DOTA for Tissue-Specific Local Inflammation Induction Monitored by FDG

C57BL/6 mice (8–12 weeks, *n* = 4 female and *n* = 4 male, from UCLA Radiation Oncology Colony) were allowed to fast overnight before being anesthetized with 1.5% vaporized isoflurane and injected ^18^F-FDG (150 μCi) via i.v. injection (tail vein). After 1 h of anesthetized uptake, static μPET/μCT baseline scans were taken (10 min). The mice were then injected with either pTrMA-*co*-DOTA or LPS (1 mg/kg) via i.v. and i.p. injection, respectively, before being fasted overnight again. After 24 h of injection with the polymer or LPS, the mice were anesthetized again with 1.5% vaporized isoflurane and injected ^18^F-FDG (150 μCi) via i.v. injection (tail vein). After 1 h of anesthetized uptake, static μPET/μCT scans were taken (10 min). All μPET images were acquired with an energy window of 350–650 keV, followed by 3D histographic analysis and reconstruction using the 3D-OSEM/MAP method. Data was decay-corrected and scaled by initial imaging. Co-registered μPET/μCT images were analyzed, and MIPs were generated using AMIDE software.

#### Plasma Lifetime Study of Insulin with or without pTrMA Excipient

CD1 mice (5–6 weeks, male, *n* = 4, Charles River Laboratories) were used for these studies. A single dose of insulin (120 μg/kg) with or without pTrMA (19.1 kDa, 2 mol equiv to insulin) was administered by i.v. injection (tail vein). Blood samples were taken from the saphenous vein (30–50 μL) at 10, 20, and 40 min and by cardiac puncture after euthanasia by inhalation of CO_2_ at 60 min following administration. Blood was collected using a micropipette with ethylenediaminetetraacetic acid (ETDA)-coated tips into LoBind tubes coated with EDTA. Blood was stored on ice until separation of the plasma by centrifugation (2000*g*, 15 min). The amount of insulin in plasma samples was determined by insulin ELISA according to the manufacturer’s protocol.

#### Insulin Stability Studies with pTrMA Excipient

Insulin was dissolved at 2 mg/mL in DPBS pH 7.4. pTrMA (28.1 kDa) was dissolved at 19 mg/mL (2 mol equiv to insulin). Solutions were added 1:1 to a total volume of 100 μL in a LoBind tube for insulin and insulin + pTrMA (*n* = 3). The samples were stored at 4 °C or heated to 90 °C for 30 min in ambient light. Aliquots of each insulin sample were used directly for SDS and native PAGE as well as ThT assay. The samples were filtered (0.22 μm) to remove insulin aggregates and analyzed by RP-HPLC. ThT was prepared at 50 μM (0.0159 mg/mL) in 20 mM DPBS pH 7.4. Into a black 96-well plate, 50 μL of each sample was pipetted. To these was added 250 μL of ThT solution, and the plate was incubated at room temperature (21 °C) for 20 min. Fluorescence intensity was measured on a Tecan M1000 plate reader (λ_ex_ = 450 nm, λ_em_ = 482 nm).

SEC-MALS investigation of insulin was performed using pTrMA (26.0 kDa). Insulin was dissolved at 30 mg/mL in minimal metal-free 0.1 M hydrochloric acid, followed by 0.1 M PBS pH 7.4. The insulin solution was used to dissolve pTrMA at 30 mg/mL, and the samples were either analyzed directly or the samples were heated to 90 °C for 30 min and then analyzed by SEC-MALS with an injection volume of 100 μL.

DSC analysis was performed using insulin at 10 mg/mL in 10 mM HCl for solubility. The samples were either run with insulin alone or with pTrMA (5 mol equiv to insulin) or the same amount of trehalose as in the polymer (50 mg/mL).

#### Long-Term Insulin Stability Studies with pTrMA Excipient

Insulin was dissolved at 2 mg/mL in DPBS pH 7.4. pTrMA (10.1 kDa) was dissolved at 2.4 mg/mL (2 mol equiv to insulin). Solutions were added 1:1 for a total volume of 1.4 mL in a LoBind tube for insulin and insulin + pTrMA (*n* = 3). The samples were stored at 4 °C for 14 weeks, followed by regular sampling. The samples were filtered (0.22 μm) to remove insulin aggregates and analyzed by RP-HPLC.

### Statistical Analysis

All experimental values are reported as the mean ± SEM, and GraphPad Prism 7 (GraphPad Software, San Diego, USA) was used for statistical analyses. To assess the statistical significance of differences with the means of two groups, the unpaired, two-tailed Student’s *t*-test was conducted assuming unequal sample variance. For experiments with more than two groups, one-way analysis of variance followed by Tukey’s test was employed to compare the means and determine the significance of the differences. Results were considered significantly different if *p* < 0.05 (*); results are also reported with *p* < 0.01 (**) and *p* < 0.001 (***).

## Supporting Information Available

Small molecule and polymer NMR spectra, GPC chromatograms, SDS-PAGE gels, DSC chromatograms, immunogenicity ELISA serum dilutions, and full PET images (PDF)

## References

[ref1] World Health Organization Ten Threats to Global Health in 2019. https://www.who.int/news-room/spotlight/ten-threats-to-global-health-in-2019 (accessed 6/01/2020).

[ref2] Kochanek K. D.; Xu J.; Arias E. Mortality in the United States, 2019; U.S. Department of Health and Human Services Centers for Disease Control and Prevention, 2020.

[ref3] World Health Organization Model List of Essential Medicines, 21st List; World Health Organization: Geneva, 2019.

[ref4] Lendner P. Economic Benefits of Improved Insulin Stability In Insulin Pumps. https://www.managedcaremag.com/archives/1105/1105-peer_insulinpump/ (accessed 4/26/2021).

[ref5] Brange J.; Langkjaer L.; Havelund S.; Vølund A. Chemical Stability of Insulin. 1. Hydrolytic Degradation During Storage of Pharmaceutical Preparations. Pharm. Res. 1992, 09, 715–726. 10.1023/a:1015835017916. 1409351

[ref6] Das A.; Shah M.; Saraogi I. Molecular Aspects of Insulin Aggregation and Various Therapeutic Interventions. ACS Bio Med Chem Au 2022, 2, 205–221. 10.1021/acsbiomedchemau.1c00054. PMC10114644 37101572

[ref7] Sluzky V.; Klibanov A. M.; Langer R. Mechanism of Insulin Aggregation and Stabilization in Agitated Aqueous Solutions. Biotechnol. Bioeng. 1992, 40, 895–903. 10.1002/bit.260400805. 18601196

[ref8] Iannuzzi C.; Borriello M.; Portaccio M.; Irace G.; Sirangelo I. Insights into Insulin Fibril Assembly at Physiological and Acidic pH and Related Amyloid Intrinsic Fluorescence. Int. J. Mol. Sci. 2017, 18, 2551 10.3390/ijms18122551. PMC5751154 29182566

[ref9] Hermeling S.; Crommelin D. J. A.; Schellekens H.; Jiskoot W. Structure-Immunogenicity Relationships of Therapeutic Proteins. Pharm. Res. 2004, 21, 897–903. 10.1023/b:pham.0000029275.41323.a6. 15212151

[ref10] Brange J.; Havelund S.; Hommel E.; Sørensen E.; Kühl C. Neutral Insulin Solutions Physically Stabilized by Addition of Zn2+. Diabetic Med. 1986, 3, 532–536. 10.1111/j.1464-5491.1986.tb00809.x. 2951208

[ref11] Brewster M. E.; Hora M. S.; Simpkins J. W.; Bodor N. Use of 2-Hydroxypropyl-β-cyclodextrin as a Solubilizing and Stabilizing Excipient for Protein Drugs. Pharm. Res. 1991, 08, 792–795. 10.1023/a:1015870521744. 2062811

[ref12] Lee H. H.; Choi T. S.; Lee S. J. C.; Lee J. W.; Park J.; Ko Y. H.; Kim W. J.; Kim K.; Kim H. I. Supramolecular Inhibition of Amyloid Fibrillation by Cucurbit[7]uril. Angew. Chem., Int. Ed. 2014, 53, 7461–7465. 10.1002/anie.201402496. 24841324

[ref13] Kitagawa K.; Misumi Y.; Ueda M.; Hayashi Y.; Tasaki M.; Obayashi K.; Yamashita T.; Jono H.; Arima H.; Ando Y. Inhibition of Insulin Amyloid Fibril Formation by Cyclodextrins. Amyloid 2015, 22, 181–186. 10.3109/13506129.2015.1064818. 26204452

[ref14] Webber M. J.; Appel E. A.; Vinciguerra B.; Cortinas A. B.; Thapa L. S.; Jhunjhunwala S.; Isaacs L.; Langer R.; Anderson D. G. Supramolecular PEGylation of Biopharmaceuticals. Proc. Natl. Acad. Sci. U.S.A. 2016, 113, 14189–14194. 10.1073/pnas.1616639113. 27911829 PMC5167179

[ref15] Maikawa C. L.; Smith A. A. A.; Zou L.; Meis C. M.; Mann J. L.; Webber M. J.; Appel E. A. Stable Monomeric Insulin Formulations Enabled by Supramolecular PEGylation of Insulin Analogues. Adv. Ther. 2020, 3, 1900094 10.1002/adtp.201900094. PMC7079736 32190729

[ref16] Meis C. M.; Salzman E. E.; Maikawa C. L.; Smith A. A. A.; Mann J. L.; Grosskopf A. K.; Appel E. A. Self-Assembled, Dilution-Responsive Hydrogels for Enhanced Thermal Stability of Insulin Biopharmaceuticals. ACS Biomater. Sci. Eng. 2020, 7, 4221–4229. 10.1021/acsbiomaterials.0c01306. 34510910 PMC8441967

[ref17] Mann J. L.; Maikawa C. L.; Smith A. A. A.; Grosskopf A. K.; Baker S. W.; Roth G. A.; Meis C. M.; Gale E. C.; Liong C. S.; Correa S.; Chan D.; Stapleton L. M.; Yu A. C.; Muir B.; Howard S.; Postma A.; Appel E. A. An Ultrafast Insulin Formulation Enabled by High-Throughput Screening of Engineered Polymeric Excipients. Sci. Transl. Med. 2020, 12, eaba6676 10.1126/scitranslmed.aba6676. 32611683 PMC7716884

[ref18] Das A.; Gangarde Y. M.; Tomar V.; Shinde O.; Upadhyay T.; Alam S.; Ghosh S.; Chaudhary V.; Saraogi I. Small-Molecule Inhibitor Prevents Insulin Fibrillogenesis and Preserves Activity. Mol. Pharm. 2020, 17, 1827–1834. 10.1021/acs.molpharmaceut.9b01080. 32347728

[ref19] Ratha B. N.; Ghosh A.; Brender J. R.; Gayen N.; Ilyas H.; Neeraja C.; Das K. P.; Mandal A. K.; Bhunia A. Inhibition of Insulin Amyloid Fibrillation by a Novel Amphipathic Heptapeptide. J. Biol. Chem. 2016, 291, 23545–23556. 10.1074/jbc.m116.742460. 27679488 PMC5095409

[ref20] Mukherjee M.; Jana J.; Chatterjee S. A Small Molecule Impedes Insulin Fibrillation: Another New Role of Phenothiazine Derivatives. ChemistryOpen 2018, 7, 68–79. 10.1002/open.201700131. 29318099 PMC5754551

[ref21] Mukherjee M.; Das D.; Sarkar J.; Banerjee N.; Jana J.; Bhat J.; Reddy G J.; Bharatam J.; Chattopadhyay S.; Chatterjee S.; Chakrabarti P. Prion-Derived Tetrapeptide Stabilizes Thermolabile Insulin via Conformational Trapping. iScience 2021, 24, 102573 10.1016/j.isci.2021.102573. 34142060 PMC8184657

[ref22] Lee J.; Lin E. W.; Lau U. Y.; Hedrick J. L.; Bat E.; Maynard H. D. Trehalose Glycopolymers as Excipients for Protein Stabilization. Biomacromolecules 2013, 14, 2561–2569. 10.1021/bm4003046. 23777473 PMC3973403

[ref23] Lee J.; Ko J. H.; Lin E. W.; Wallace P.; Ruch F.; Maynard H. D. Trehalose Hydrogels for Stabilization of Enzymes to Heat. Polym. Chem. 2015, 6, 3443–3448. 10.1039/c5py00121h. 26005500 PMC4436147

[ref24] Lau U. Y.; Saxer S. S.; Lee J.; Bat E.; Maynard H. D. Direct Write Protein Patterns for Multiplexed Cytokine Detection from Live Cells Using Electron Beam Lithography. ACS Nano 2016, 10, 723–729. 10.1021/acsnano.5b05781. 26679368 PMC4729597

[ref25] Pelegri-O’Day E. M.; Paluck S. J.; Maynard H. D. Substituted Polyesters by Thiol-Ene Modification: Rapid Diversification for Therapeutic Protein Stabilization. J. Am. Chem. Soc. 2017, 139, 1145–1154. 10.1021/jacs.6b10776. 28079370 PMC5509517

[ref26] Messina M. S.; Ko J. H.; Yang Z. Y.; Strouse M. J.; Houk K. N.; Maynard H. D. Effect of Trehalose Polymer Regioisomers on Protein Stabilization. Polym. Chem. 2017, 8, 4781–4788. 10.1039/c7py00700k.

[ref27] Liu Y.; Lee J.; Mansfield K. M.; Ko J. H.; Sallam S.; Wesdemiotis C.; Maynard H. D. Trehalose Glycopolymer Enhances Both Solution Stability and Pharmacokinetics of a Therapeutic Protein. Bioconjugate Chem. 2017, 28, 836–845. 10.1021/acs.bioconjchem.6b00659. PMC5352466 28044441

[ref28] Mansfield K. M.; Maynard H. D. Site-Specific Insulin-Trehalose Glycopolymer Conjugate by Grafting from Strategy Improves Bioactivity. ACS Macro Lett. 2018, 7, 324–329. 10.1021/acsmacrolett.7b00974. 30467526 PMC6241536

[ref29] Lee J.; Ko J. H.; Mansfield K. M.; Nauka P. C.; Bat E.; Maynard H. D. Glucose-Responsive Trehalose Hydrogel for Insulin Stabilization and Delivery. Macromol. Biosci. 2018, 18, 1700372 10.1002/mabi.201700372. PMC5986559 29665232

[ref30] Pelegri-O’Day E. M.; Bhattacharya A.; Theopold N.; Ko J. H.; Maynard H. D. Synthesis of Zwitterionic and Trehalose Polymers with Variable Degradation Rates and Stabilization of Insulin. Biomacromolecules 2020, 21, 2147–2154. 10.1021/acs.biomac.0c00133. 32369347 PMC8259896

[ref31] Gelb M. B.; Maynard H. D. Effect of Poly(trehalose methacrylate) Molecular Weight and Concentration on the Stability and Viscosity of Insulin. Macromol. Mater. Eng. 2021, 306, 2100197 10.1002/mame.202100197. 35591895 PMC9113406

[ref32] Dias C.; Abosaleem B.; Crispino C.; Gao B.; Shaywitz A. Tolerability of High-Volume Subcutaneous Injections of a Viscous Placebo Buffer: A Randomized, Crossover Study in Healthy Subjects. AAPS PharmSciTech. 2015, 16, 1101–1107. 10.1208/s12249-015-0288-y. 25693652 PMC4674646

[ref33] Ko J. H.; Forsythe N. L.; Gelb M. B.; Messina K. M. M.; Lau U. Y.; Bhattacharya A.; Olafsen T.; Lee J. T.; Kelly K. A.; Maynard H. D. Safety and Biodistribution Profile of Poly(styrenyl acetal trehalose) and Its Granulocyte Colony Stimulating Factor Conjugate. Biomacromolecules 2022, 23, 3383–3395. 10.1021/acs.biomac.2c00511. 35767465

[ref34] Cataldi M.; Vigliotti C.; Mosca T.; Cammarota M.; Capone D. Emerging Role of the Spleen in the Pharmacokinetics of Monoclonal Antibodies, Nanoparticles and Exosomes. Int. J. Mol. Sci. 2017, 18, 1249 10.3390/ijms18061249. PMC5486072 28604595

[ref35] Attri A. K.; Fernández C.; Minton A. P. Self-Association of Zn-Insulin at Neutral pH: Investigation by Concentration Gradient-Static and Dynamic Light Scattering. Biophys. Chem. 2010, 148, 23–7. 10.1016/j.bpc.2010.02.001. 20202736 PMC2867077

[ref36] Jonassen I.; Havelund S.; Hoeg-Jensen T.; Steensgaard D. B.; Wahlund P.-O.; Ribel U. Design of the Novel Protraction Mechanism of Insulin Degludec, an Ultra-long-Acting Basal Insulin. Pharm. Res. 2012, 29, 2104–2114. 10.1007/s11095-012-0739-z. 22485010 PMC3399081

[ref37] Frazer R. Q.; Byron R. T.; Osborne P. B.; West K. P. PMMA: An Essential Material in Medicine and Dentistry. J. Long-Term Eff. Med. Implants 2005, 15, 629–639. 10.1615/jlongtermeffmedimplants.v15.i6.60. 16393131

[ref38] Samavedi S.; Poindexter L. K.; Van Dyke M.; Goldstein A. S. Synthetic Biomaterials for Regenerative Medicine Applications. In Regenerative Medicine Applications in Organ Transplantation; Orlando G., Lerut J., Soker S., Stratta R. J., Eds.; Academic Press: Boston, 2014; Chapter 7, pp 81–99.

[ref39] Jiang L.; Tu Y.; Hu X.; Bao A.; Chen H.; Ma X.; Doyle T.; Shi H.; Cheng Z. Pilot Study of 64Cu(I) for PET Imaging of Melanoma. Sci. Rep. 2017, 7, 2574 10.1038/s41598-017-02691-3. 28566692 PMC5451408

[ref40] Šprincl L.; Exner J.; Štěrba O.; Kopeček J. New Types of Synthetic Infusion Solutions. III. Elimination and Retention of Poly-[N-(2-hydroxypropyl)methacrylamide] in a Test Organism. J. Biomed. Mater. Res. 1976, 10, 953–963. 10.1002/jbm.820100612. 993230

[ref41] Sun X.; Rossin R.; Turner J. L.; Becker M. L.; Joralemon M. J.; Welch M. J.; Wooley K. L. An Assessment of the Effects of Shell Cross-Linked Nanoparticle Size, Core Composition, and Surface PEGylation on in Vivo Biodistribution. Biomacromolecules 2005, 6, 2541–2554. 10.1021/bm050260e. 16153091 PMC2533516

[ref42] Pressly E. D.; Rossin R.; Hagooly A.; Fukukawa K.-i.; Messmore B. W.; Welch M. J.; Wooley K. L.; Lamm M. S.; Hule R. A.; Pochan D. J.; Hawker C. J. Structural Effects on the Biodistribution and Positron Emission Tomography (PET) Imaging of Well-Defined 64Cu-Labeled Nanoparticles Comprised of Amphiphilic Block Graft Copolymers. Biomacromolecules 2007, 8, 3126–3134. 10.1021/bm700541e. 17880180

[ref43] Fukukawa K.-i.; Rossin R.; Hagooly A.; Pressly E. D.; Hunt J. N.; Messmore B. W.; Hawker K. L.; Welch M. J.; Hawker C. J. Synthesis and Characterization of Core–Shell Star Copolymers for In Vivo PET Imaging Applications. Biomacromolecules 2008, 9, 1329–1339. 10.1021/bm7014152. 18338840

[ref44] Welch M. J.; Hawker C. J.; Wooley K. L. The Advantages of Nanoparticles for PET. J. Nucl. Med. 2009, 50, 1743–1746. 10.2967/jnumed.109.061846. 19837751

[ref45] Sun G.; Hagooly A.; Xu J.; Nyström A. M.; Li Z.; Rossin R.; Moore D. A.; Wooley K. L.; Welch M. J. Facile, Efficient Approach to Accomplish Tunable Chemistries and Variable Biodistributions for Shell Cross-Linked Nanoparticles. Biomacromolecules 2008, 9, 1997–2006. 10.1021/bm800246x. 18510359

[ref46] Correia M.; Neves-Petersen M. T.; Jeppesen P. B.; Gregersen S.; Petersen S. B. UV-Light Exposure of Insulin: Pharmaceutical Implications upon Covalent Insulin Dityrosine Dimerization and Disulphide Bond Photolysis. PLoS One 2012, 7, e50733 10.1371/journal.pone.0050733. 23227203 PMC3515625

[ref47] Mozziconacci O.; Haywood J.; Gorman E. M.; Munson E.; Schöneich C. Photolysis of Recombinant Human Insulin in the Solid State: Formation of a Dithiohemiacetal Product at the C-Terminal Disulfide Bond. Pharm. Res. 2012, 29, 121–133. 10.1007/s11095-011-0519-1. 21748537

[ref48] Manning M. C.; Chou D. K.; Murphy B. M.; Payne R. W.; Katayama D. S. Stability of Protein Pharmaceuticals: An Update. Pharm. Res. 2010, 27, 544–575. 10.1007/s11095-009-0045-6. 20143256

[ref49] Boehnke N.; Kammeyer J. K.; Damoiseaux R.; Maynard H. D. Stabilization of Glucagon by Trehalose Glycopolymer Nanogels. Adv. Funct. Mater. 2018, 28, 1705475 10.1002/adfm.201705475.

[ref50] Ren L.; Zhang J.; Hardy C. G.; Doxie D.; Fleming B.; Tang C. Preparation of Cobaltocenium-Labeled Polymers by Atom Transfer Radical Polymerization. Macromolecules 2012, 45, 2267–2275. 10.1021/ma202725c.

[ref51] Jin J.; Liu J.; Lian X.; Sun P.; Zhao H. Dynamic Polymer Brushes on the Surface of Silica Particles. RSC Adv. 2013, 3, 7023–7029. 10.1039/c3ra40227d.

