# Supplementary material for: Poly(trehalose methacrylate) as an Excipient for Insulin Stabilization: Mechanism and Safety
Source: ACS Appl Mater Interfaces. 2022 Aug 15;14(33):37410–23. doi: 10.1021/acsami.2c09301 (PMC9412841; doi:10.1021/acsami.2c09301)
Supplement: Supplementary file 1 — am2c09301_si_001.pdf [file am2c09301_si_001.pdf]

Supporting Information

Poly(trehalose methacrylate) as an Excipient for Insulin Stabilization: Mechanism and Safety

Madeline B. Gelb[†], Kathryn M. M. Messina[†], Daniele Vinciguerra[†], Jeong Hoon Ko[†] Jeffrey Collins[‡], Mikayla Tamboline[‡], Shili Xu[‡], F. Javier Ibarrondo[§], Heather D. Maynard*[†].

[†]Department of Chemistry and Biochemistry and California NanoSystems Institute, University of California, Los Angeles, 607 Charles E. Young Drive East, Los Angeles, California 90095-1569, USA

[‡]Department of Molecular and Medical Pharmacology and Crump Institute for Molecular Imaging, David Geffen School of Medicine, University of California, Los Angeles, CA, USA

[§]Division of Infectious Diseases, Department of Medicine, David Geffen School of Medicine at UCLA, Los Angeles, California 90095-1569, USA

*Corresponding author email: maynard@chem.ucla.edu

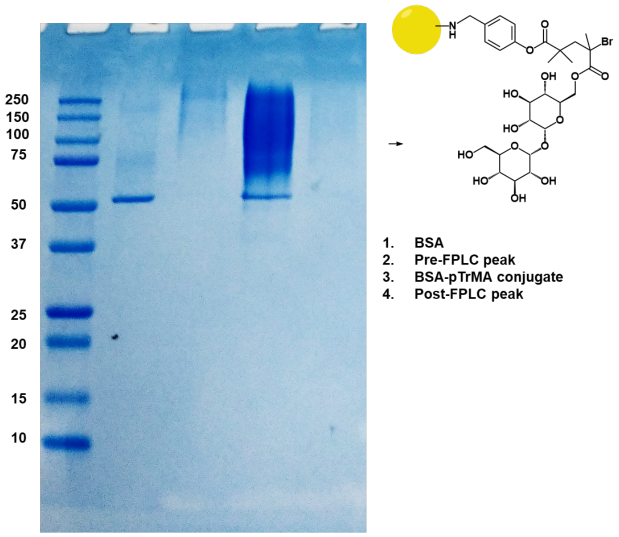

Figure S1. SDS-PAGE gel of BSA-pTrMA conjugate after purifying by FPLC and combining the pre-, post-, and conjugate peak containing fractions.

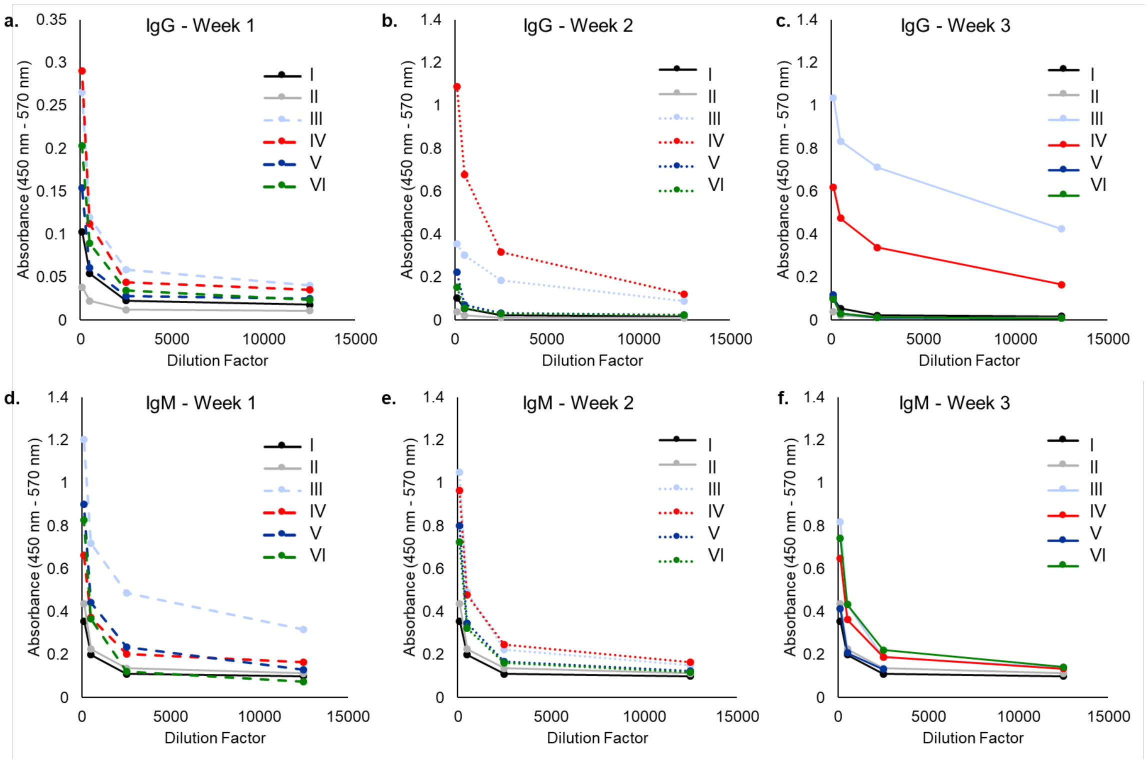

Figure S2. Immunogenicity ELISA serum dilutions of a. IgG week 1, b. IgG week 2, c. IgG week 3, d. IgM week 1, e. IgM week 2, and f. IgM week 3 for I. naïve mouse serum with OVA antigen, II. naïve mouse serum with BSA-pTrMA antigen, III. mouse serum from mice injected with OVA with OVA antigen, IV. mouse serum from mice injected with OVA+pTrMA with OVA antigen, V. mouse serum from mice injected with OVA+pTrMA with BSA-pTrMA antigen, VI. mouse serum from mice injected with pTrMA with pTrMA antigen.

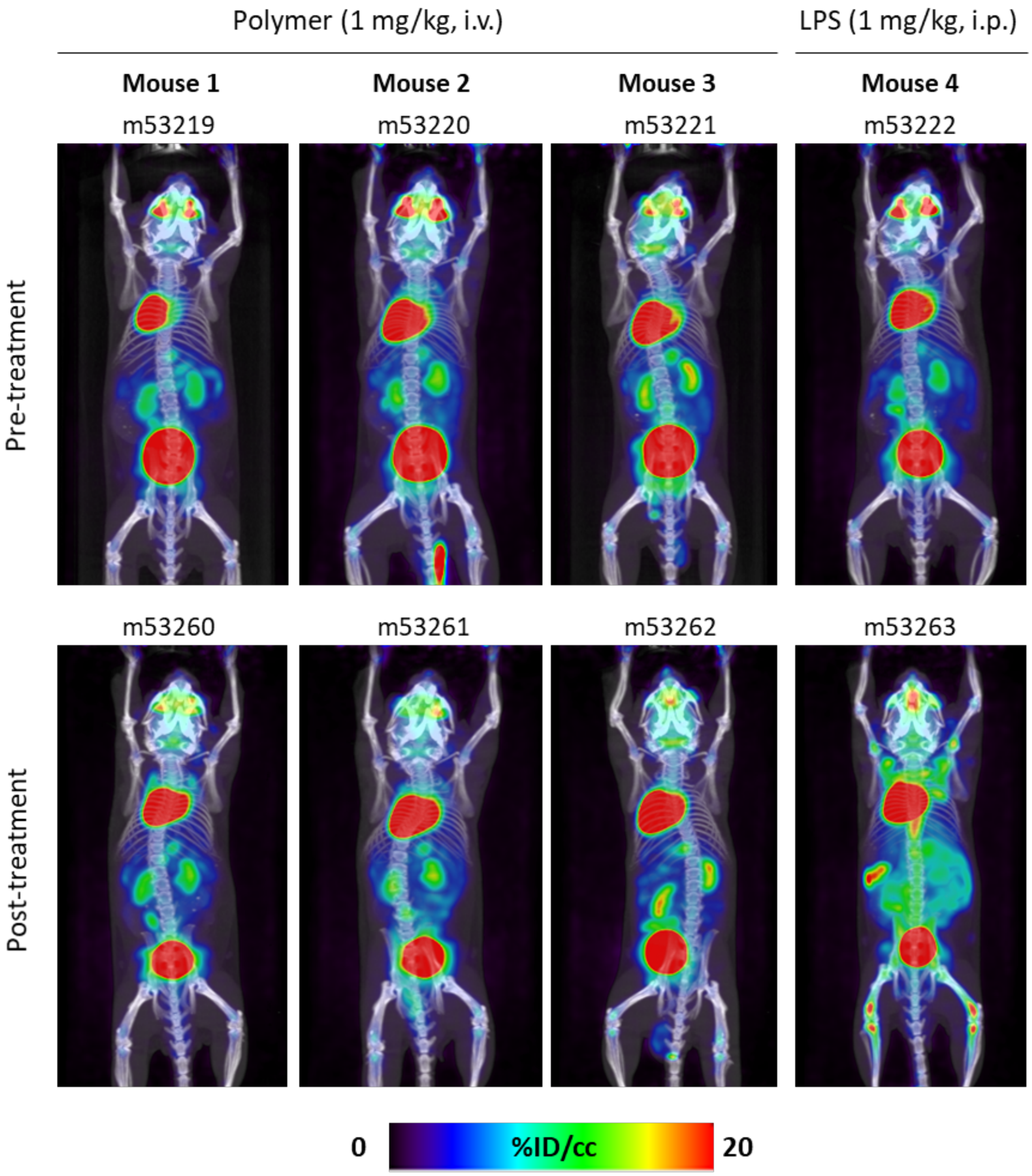

Figure S3. Co-registered μPET and μCT scans of ^{18}F-fludeoxyglucose (FDG) injection to male mice (n = 4) pre- and 24 h post- i.v. injection with pTrMA-*co*-DOTA (1 mg/kg).

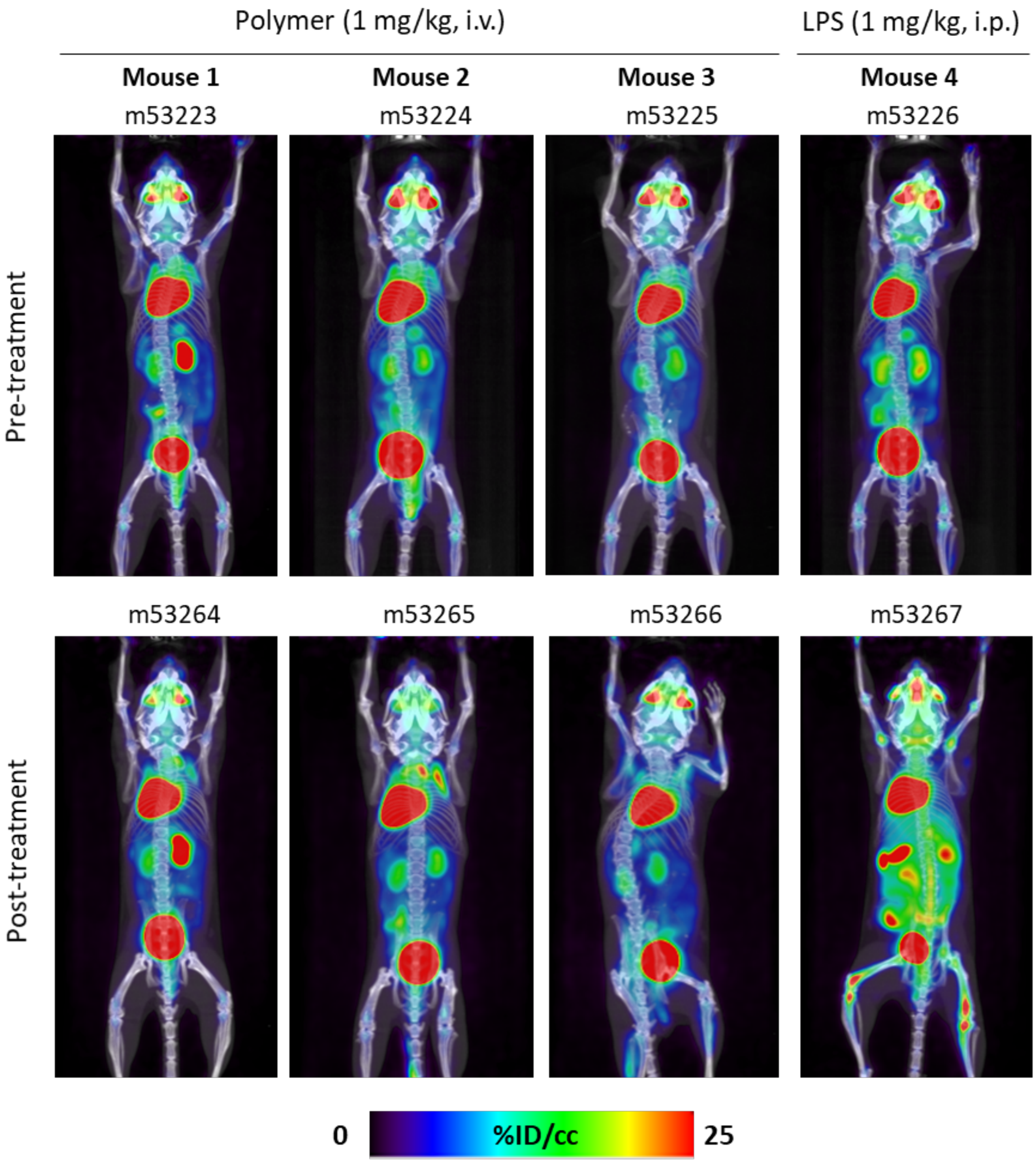

Figure S4. Co-registered µPET and µCT scans of ^{18}F-FDG injection to female mice (n = 4) pre- and 24 h post- i.p. injection with LPS (1 mg/kg).

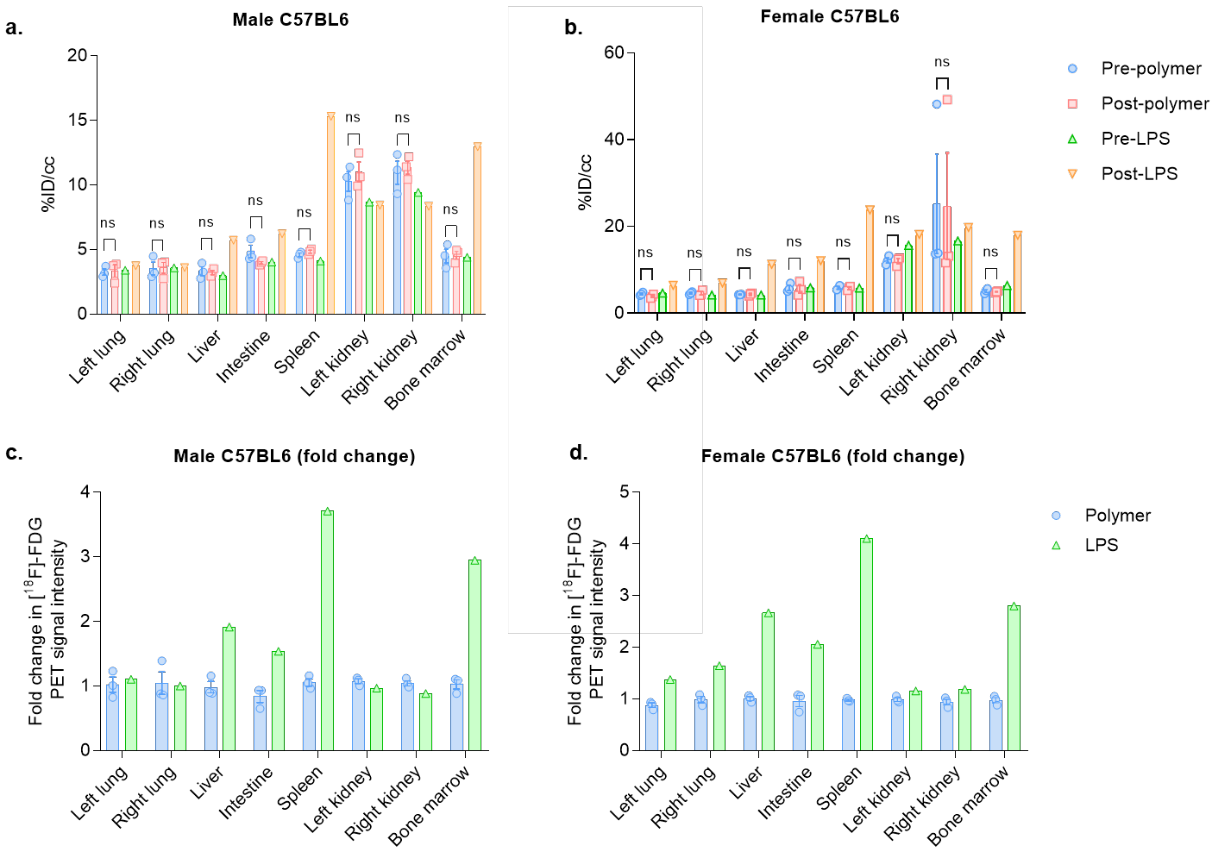

Figure S5. Quantification of µPET scans of ^{18}F-FDG (150 μCi, i.v.) injection to mice pre- and 24 h post- injection of pTrMA-*co*-DOTA or LPS (1 mg/kg, i.v. and i.p., respectively) from the organs with appreciable signal (left lung, right lung, liver, intestine, spleen, left kidney, right kidney, and bone marrow) for a) male mice and b) female mice. Fold change from before to after injection with polymer or LPS was calculated and plotted for the c. male and d. female mice.

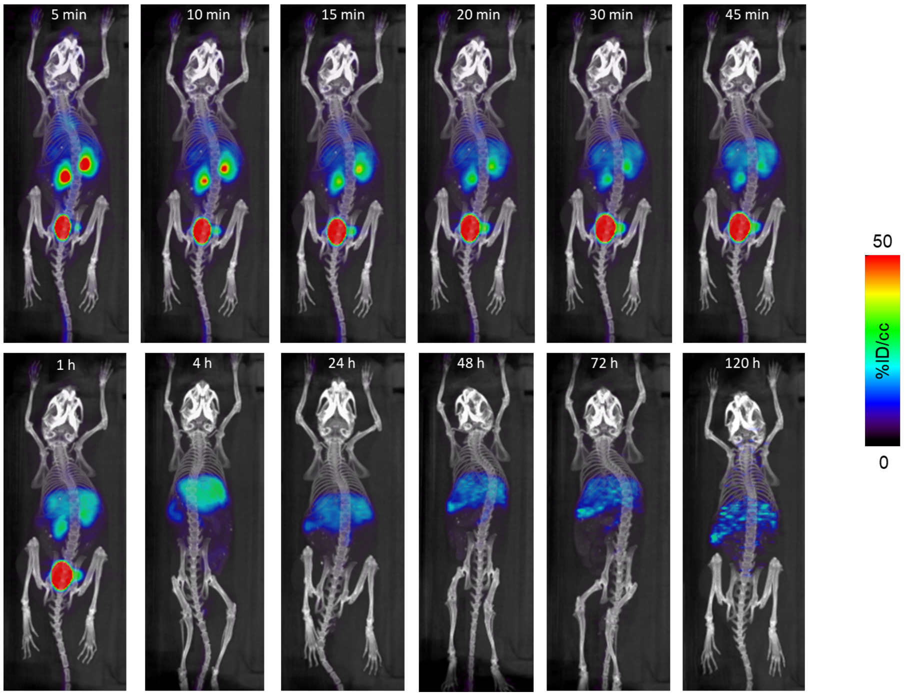

Figure S6. Co-registered μPET and μCT scans post ^{64}Cu-labeled pTrMA-*co*-DOTA injection to female mouse 1.

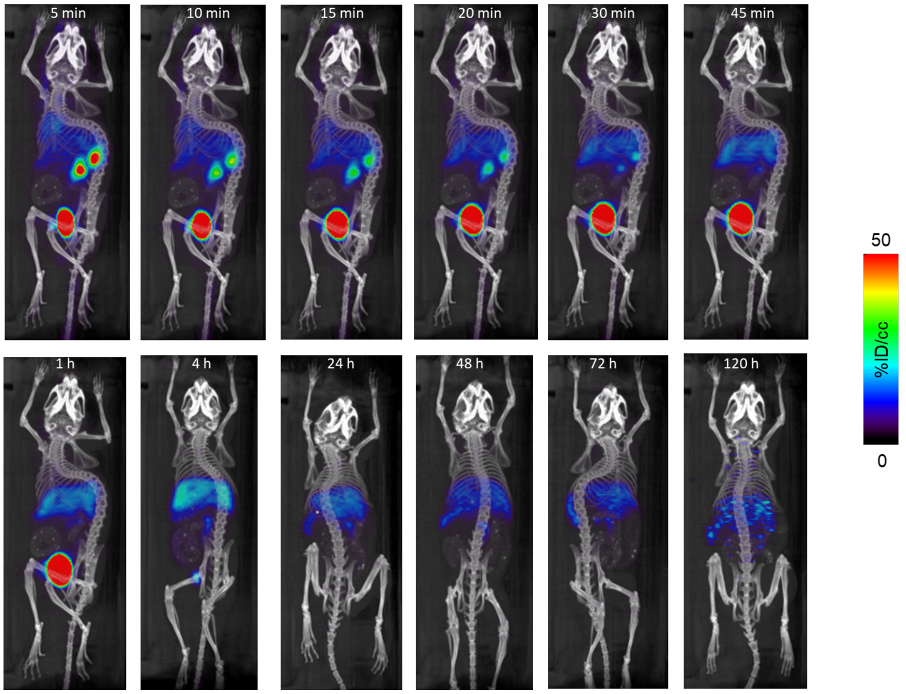

Figure S7. Co-registered μPET and μCT scans post ^{64}Cu-labeled pTrMA-*co*-DOTA injection to female mouse 2.

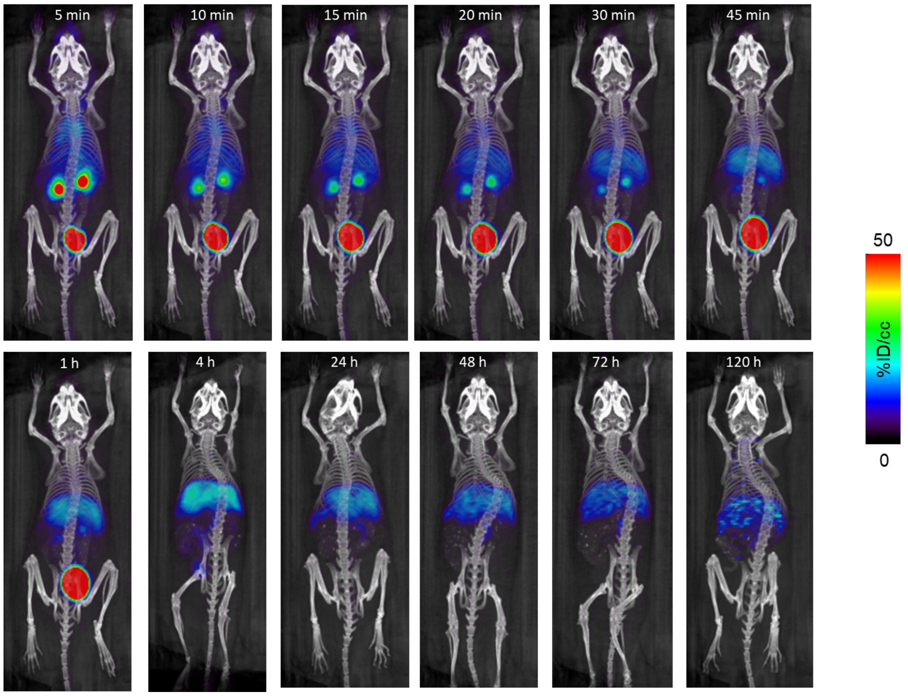

Figure S8. Co-registered µPET and µCT scans post ^{64}Cu-labeled pTrMA-*co*-DOTA injection to female mouse 3.

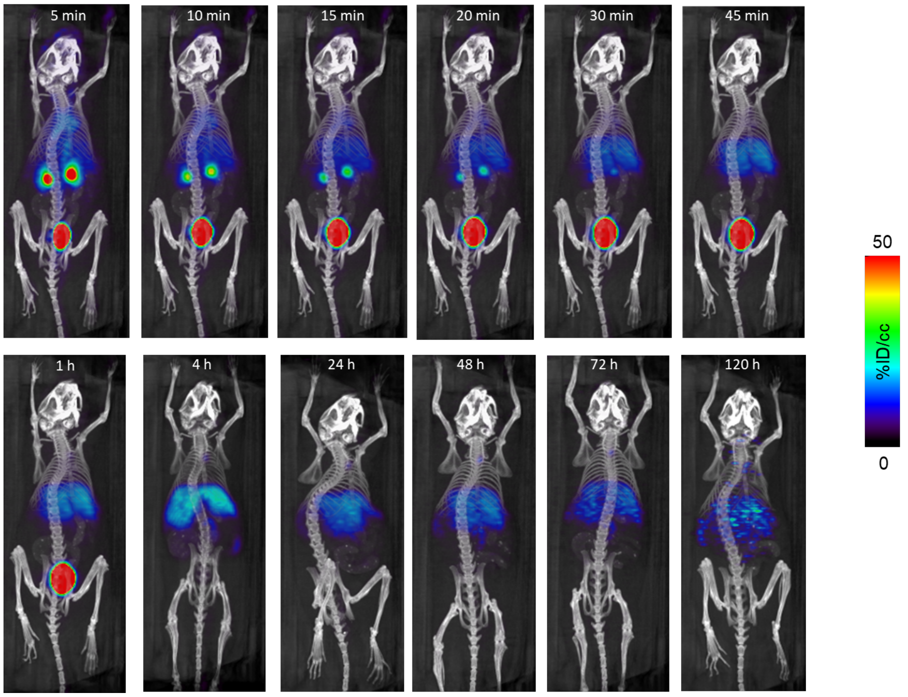

Figure S9. Co-registered µPET and µCT scans post ^{64}Cu-labeled pTrMA-*co*-DOTA injection to female mouse 4.

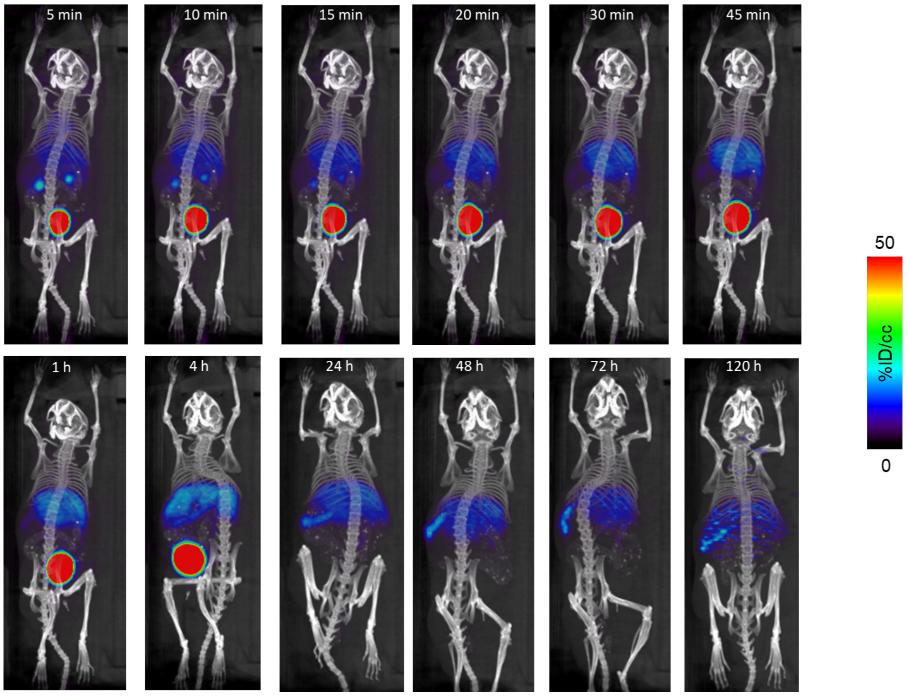

Figure S10. Co-registered µPET and µCT scans post ^{64}Cu-labeled pTrMA-*co*-DOTA injection to male mouse 1.

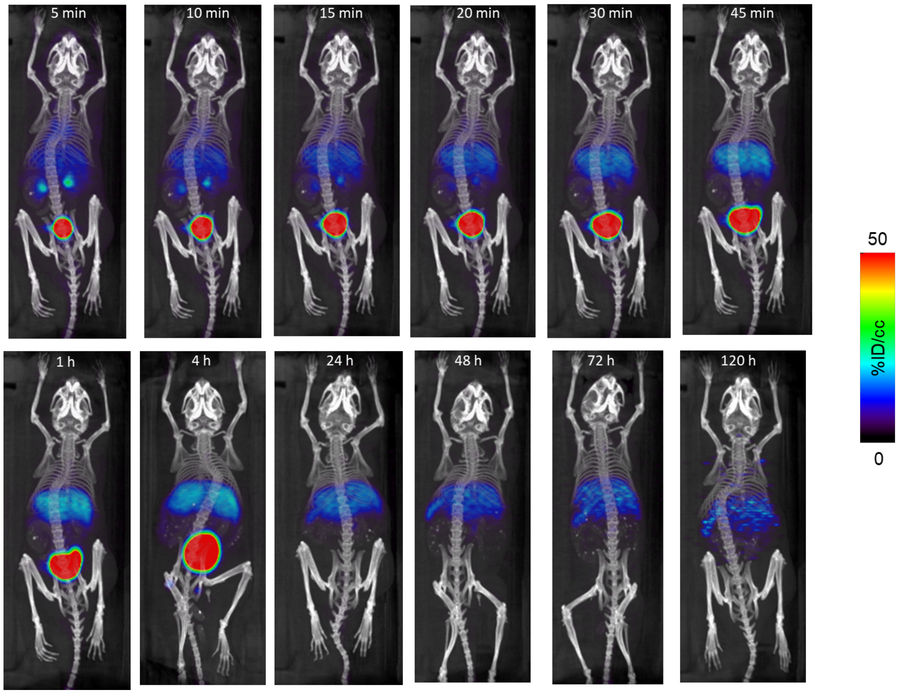

Figure S11. Co-registered µPET and µCT scans post ^{64}Cu-labeled pTrMA-*co*-DOTA injection to male mouse 2.

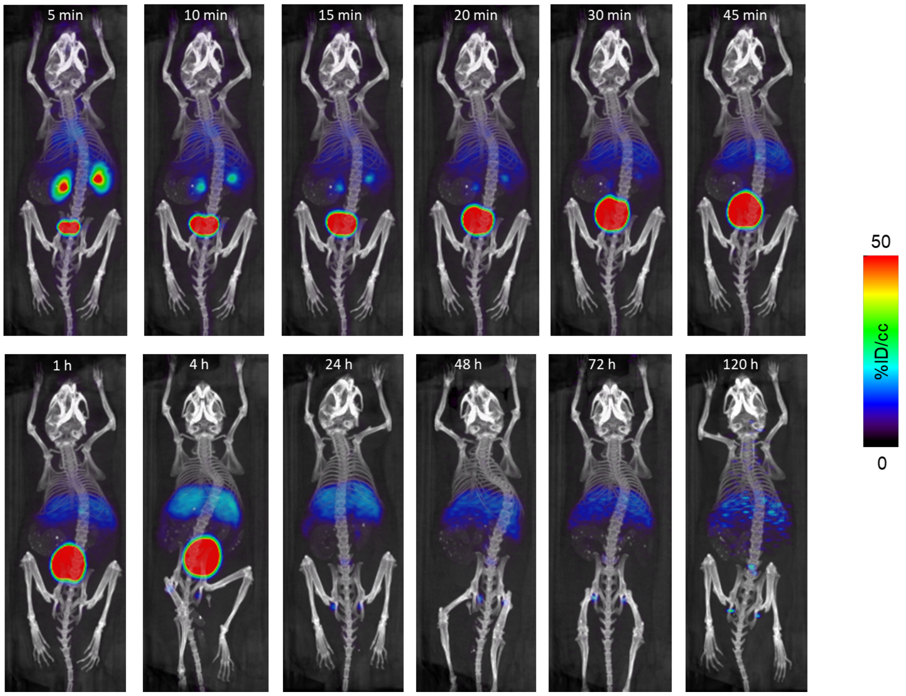

Figure S12. Co-registered μPET and μCT scans post ^{64}Cu-labeled pTrMA -*co*-DOTA injection to male mouse 3.

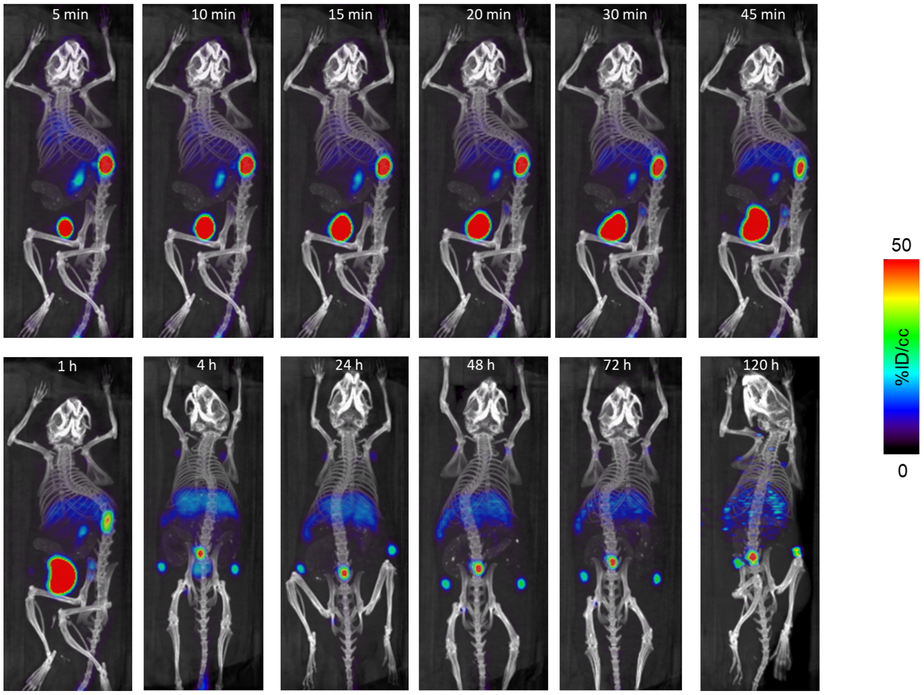

Figure S13. Co-registered μPET and μCT scans post ^{64}Cu-labeled pTrMA-*co*-DOTA injection to male mouse 4.

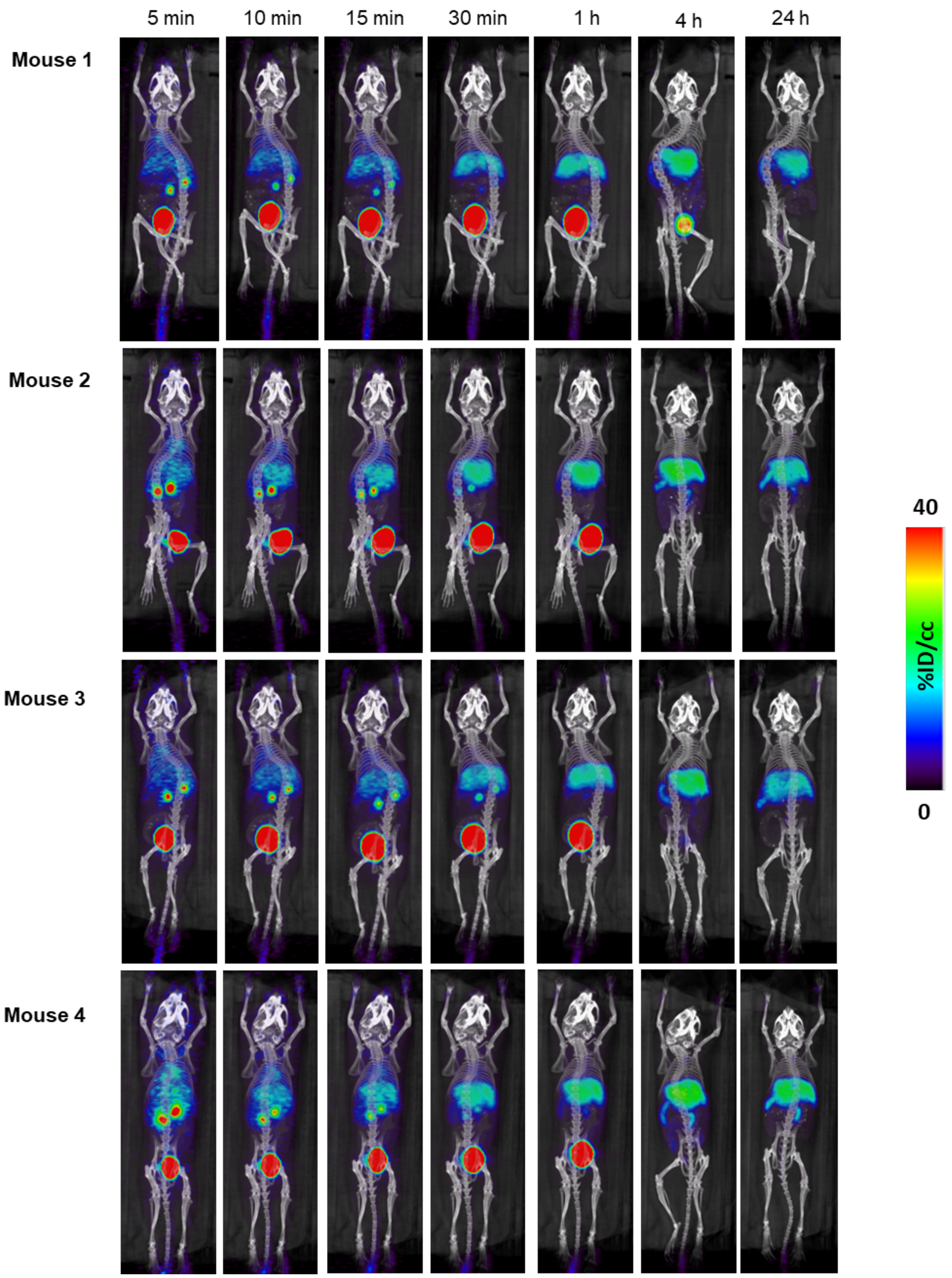

Figure S14. Co-registered μPET and μCT scans post fresh ^{64}Cu-labeled pTrMA-*co*-DOTA injection in mice ($n = 4$).

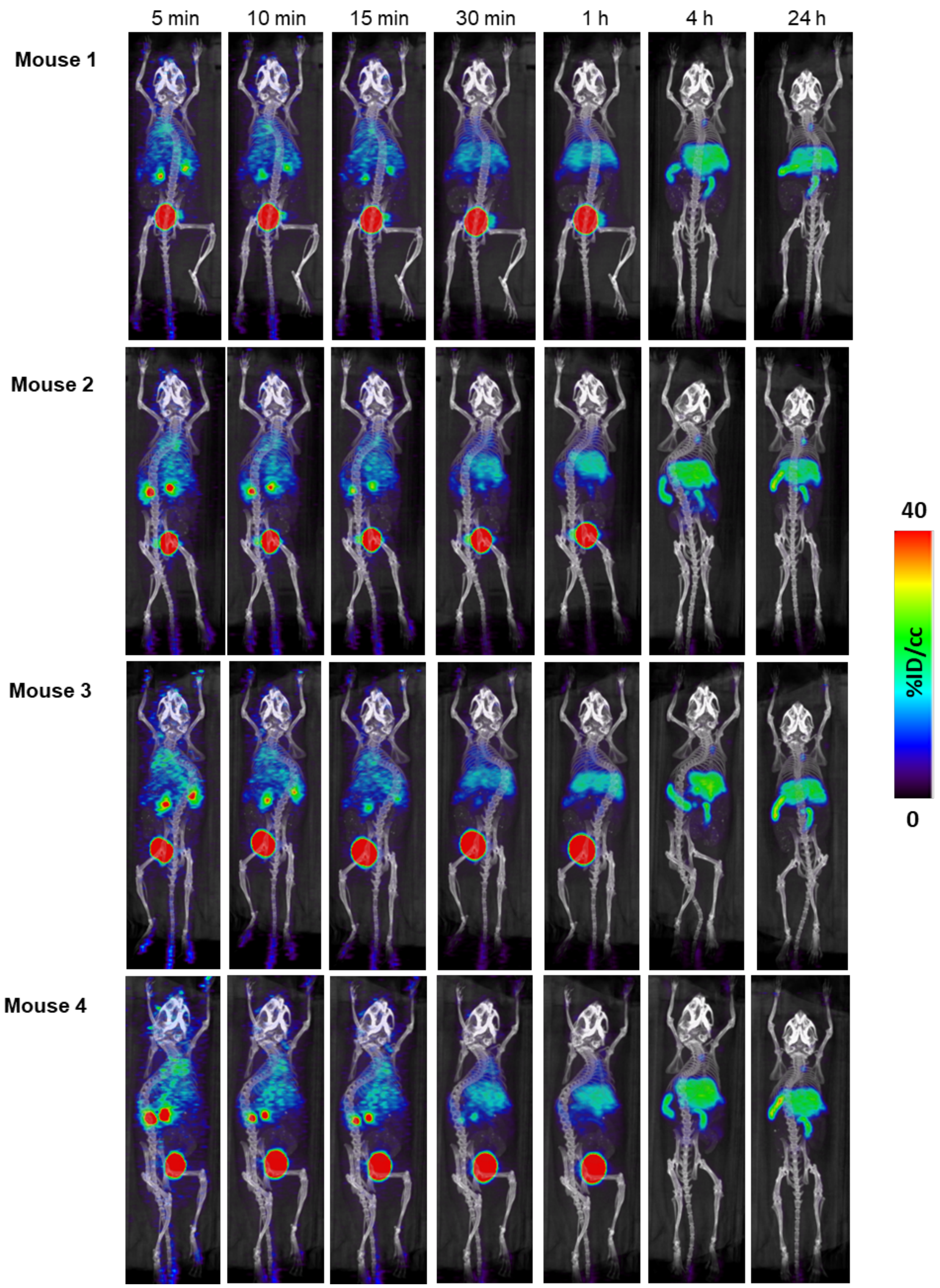

Figure S15. Co-registered μPET and μCT scans post plasma-incubated ^{64}Cu-labeled pTrMA-*co*-DOTA injection in mice (n = 4).

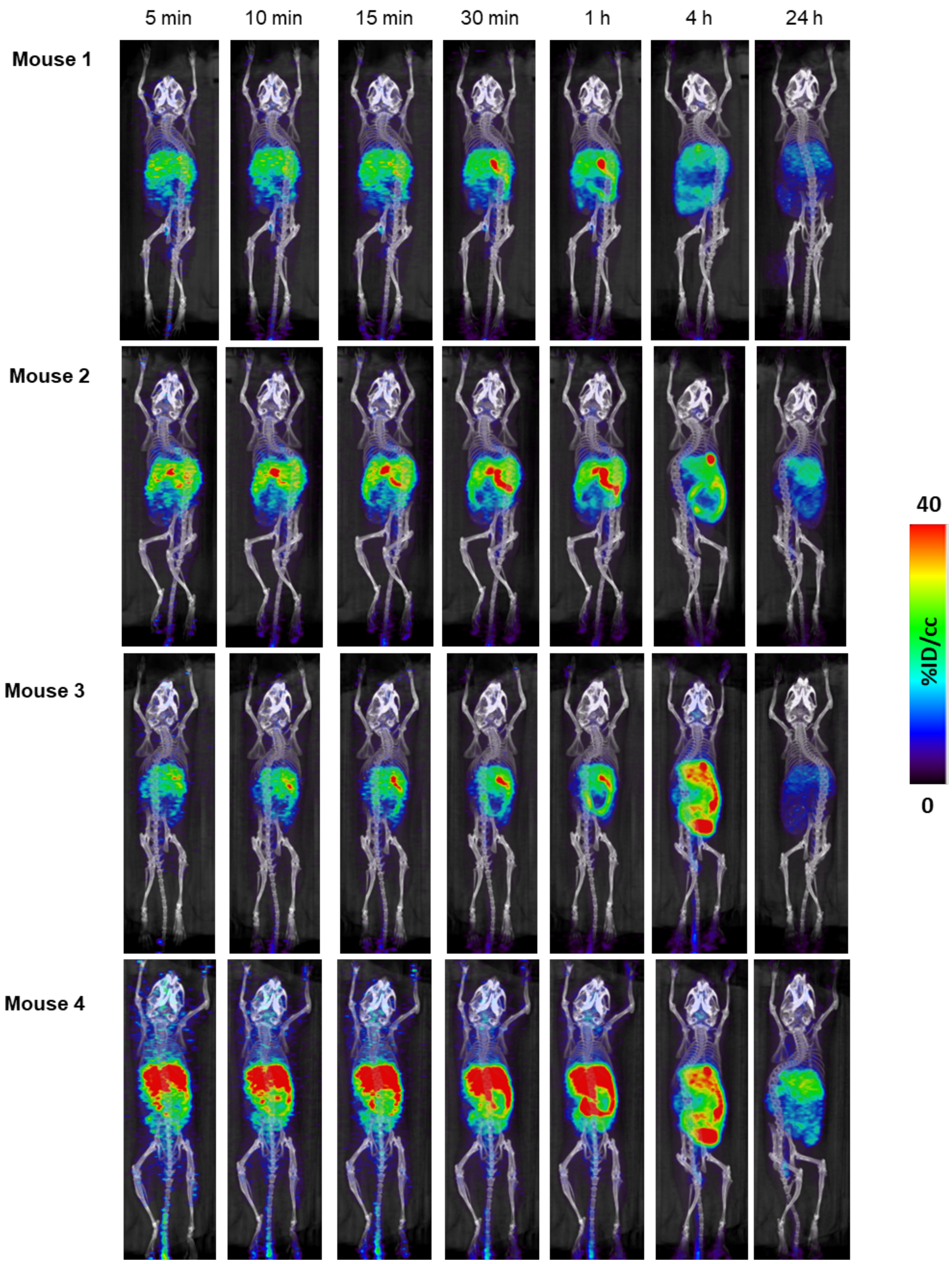

Figure S16. Co-registered μPET and μCT scans post $^{64}CuCl_2$ injection in mice (n = 4).

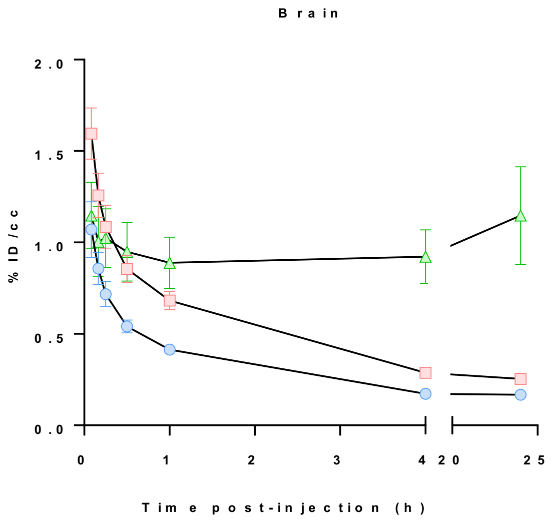

Figure S17. Time-course μPET/μCT study tracking biodistribution and excretion from the brain of female (*n* = 4) mice injected with fresh ^{64}Cu-labeled pTrMA-*co*-DOTA (blue), plasma incubated ^{64}Cu-labeled pTrMA-*co*-DOTA (red), and free $^{64}CuCl_2$ (green).

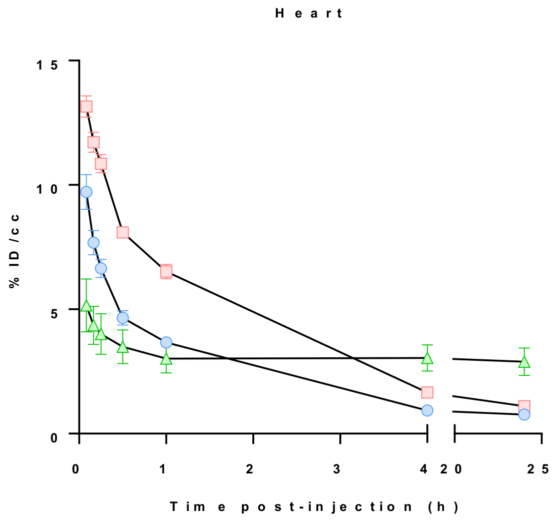

Figure S18. Time-course μPET/μCT study tracking biodistribution and excretion from the heart of female (*n* = 4) mice injected with fresh ^{64}Cu-labeled pTrMA-*co*-DOTA (blue), plasma incubated ^{64}Cu-labeled pTrMA-*co*-DOTA (red), and free $^{64}CuCl_2$ (green).

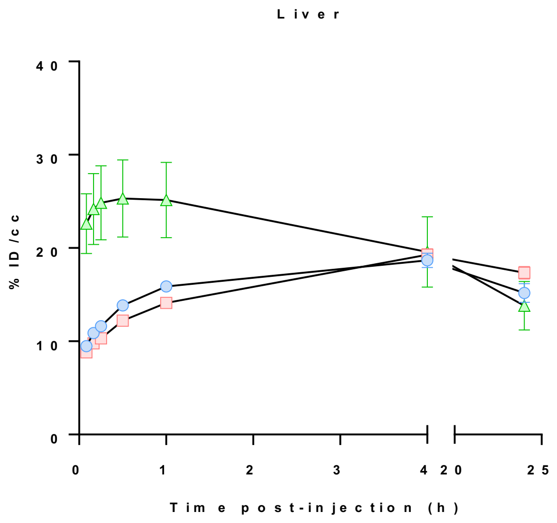

Figure S19. Time-course μPET/μCT study tracking biodistribution and excretion from the liver of female ($n = 4$) mice injected with fresh ^{64}Cu-labeled pTrMA-*co*-DOTA (blue), plasma incubated ^{64}Cu-labeled pTrMA-*co*-DOTA (red), and free $^{64}CuCl_2$ (green).

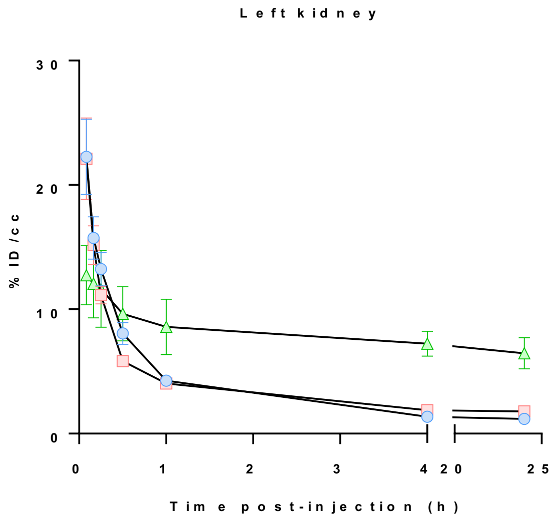

Figure S20. Time-course μPET/μCT study tracking biodistribution and excretion from the left kidney of female ($n = 4$) mice injected with fresh ^{64}Cu-labeled pTrMA-*co*-DOTA (blue), plasma incubated ^{64}Cu-labeled pTrMA-*co*-DOTA (red), and free $^{64}CuCl_2$ (green).

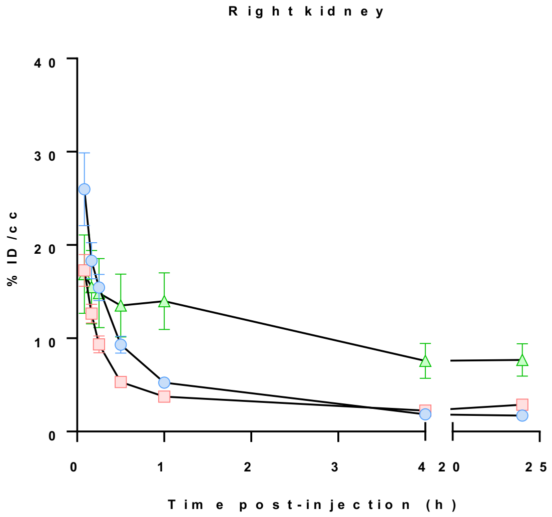

Figure S21. Time-course μPET/μCT study tracking biodistribution and excretion from the right kidney of female (n = 4) mice injected with fresh ^{64}Cu-labeled pTrMA-*co*-DOTA (blue), plasma incubated ^{64}Cu-labeled pTrMA-*co*-DOTA (red), and free $^{64}CuCl_2$ (green).

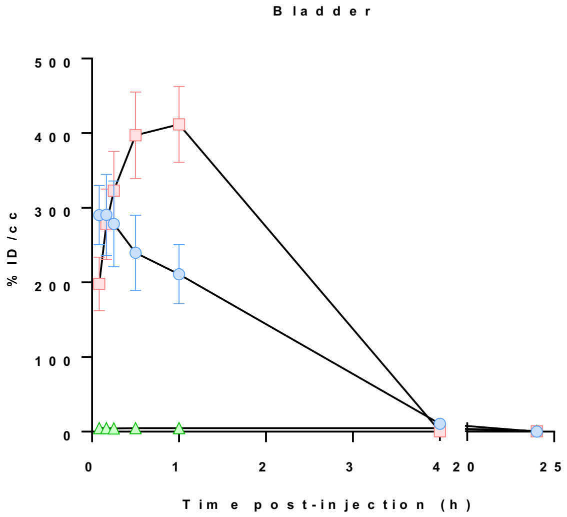

Figure S22. Time-course μPET/μCT study tracking biodistribution and excretion from the bladder of female (n = 4) mice injected with fresh ^{64}Cu-labeled pTrMA-*co*-DOTA (blue), plasma incubated ^{64}Cu-labeled pTrMA-*co*-DOTA (red), and free $^{64}CuCl_2$ (green).

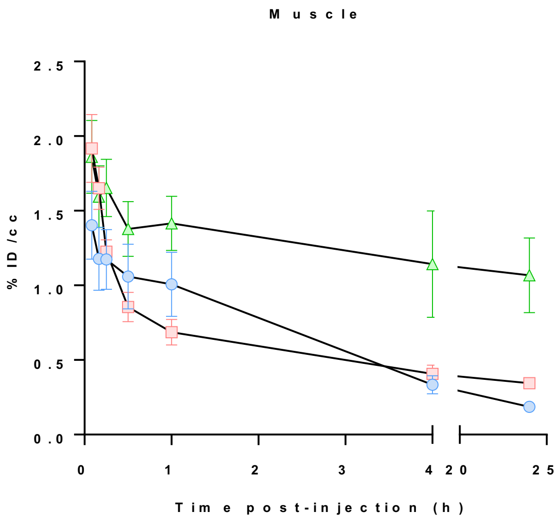

Figure S23. Time-course μPET/μCT study tracking biodistribution and excretion from the muscle of female (n = 4) mice injected with fresh ^{64}Cu-labeled pTrMA-*co*-DOTA (blue), plasma incubated ^{64}Cu-labeled pTrMA-*co*-DOTA (red), and free $^{64}CuCl_2$ (green).

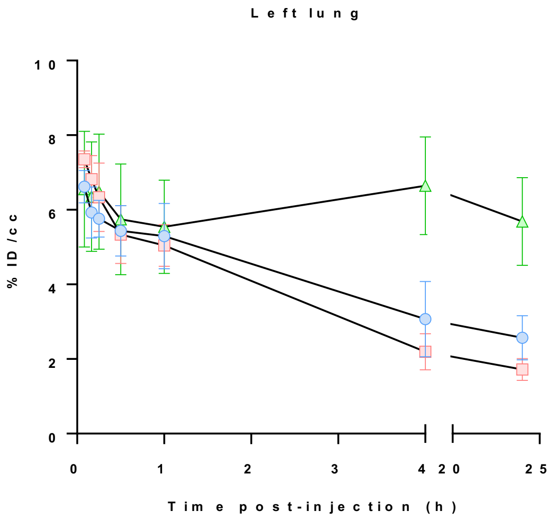

Figure S24. Time-course μPET/μCT study tracking biodistribution and excretion from the left lung of female (n = 4) mice injected with fresh ^{64}Cu-labeled pTrMA-*co*-DOTA (blue), plasma incubated ^{64}Cu-labeled pTrMA-*co*-DOTA (red), and free $^{64}CuCl_2$ (green).

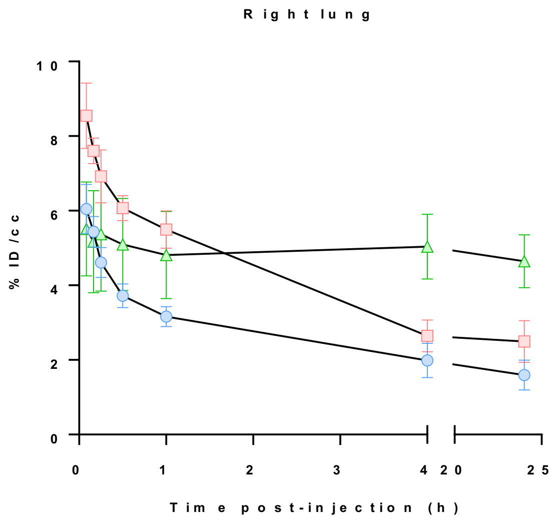

Figure S25. Time-course µPET/µCT study tracking biodistribution and excretion from the right lung of female (n = 4) mice injected with fresh ^{64}Cu-labeled pTrMA-*co*-DOTA (blue), plasma incubated ^{64}Cu-labeled pTrMA-*co*-DOTA (red), and free $^{64}CuCl_2$ (green).

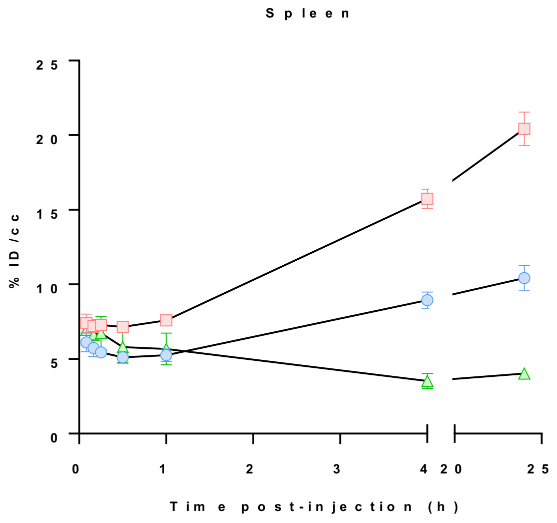

Figure S26. Time-course µPET/µCT study tracking biodistribution and excretion from the spleen of female (n = 4) mice injected with fresh ^{64}Cu-labeled pTrMA-*co*-DOTA (blue), plasma incubated ^{64}Cu-labeled pTrMA-*co*-DOTA (red), and free $^{64}CuCl_2$ (green).

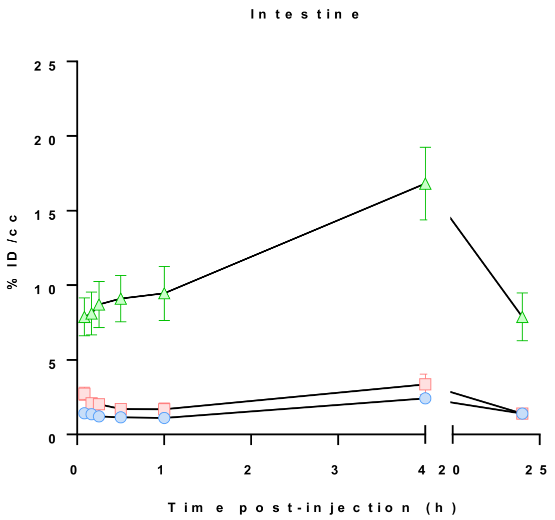

Figure S27. Time-course μPET/μCT study tracking biodistribution and excretion from the intestine of female (n = 4) mice injected with fresh ^{64}Cu-labeled pTrMA-*co*-DOTA (blue), plasma incubated ^{64}Cu-labeled pTrMA-*co*-DOTA (red), and free $^{64}CuCl_2$ (green).

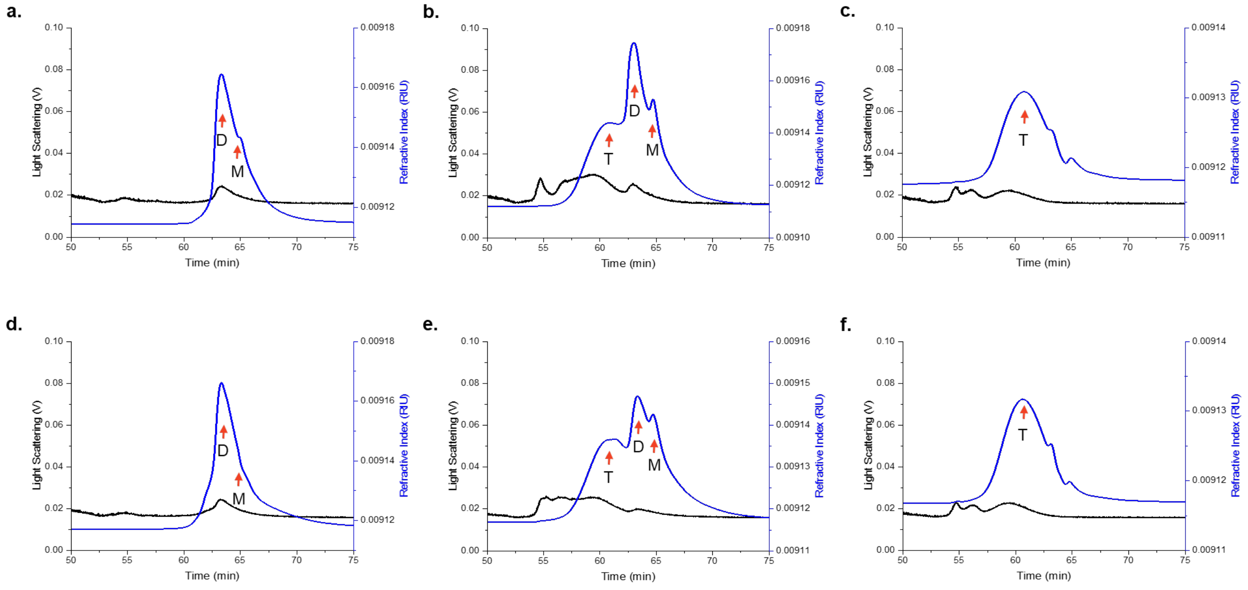

Figure S28. SEC-MALS elution profiles of a. fresh insulin, b. fresh insulin with pTrMA, c. fresh pTrMA, d. heated insulin, e. heated insulin with pTrMA, and f. heated pTrMA. Peaks are labeled as dimer (D), monomer (M), or pTrMA (T).

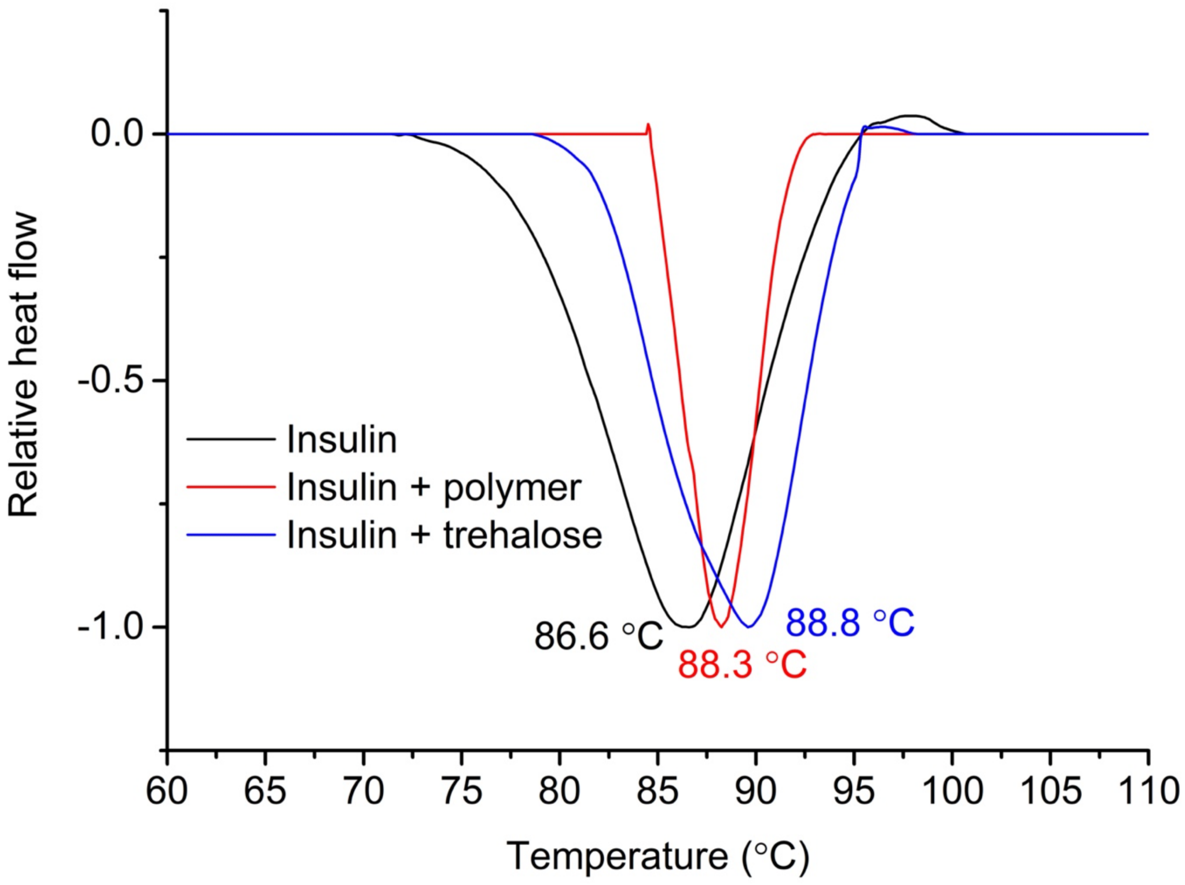

Figure S29. DSC thermograms and melting temperatures of insulin (black), insulin with pTrMA (red), insulin with trehalose (blue).

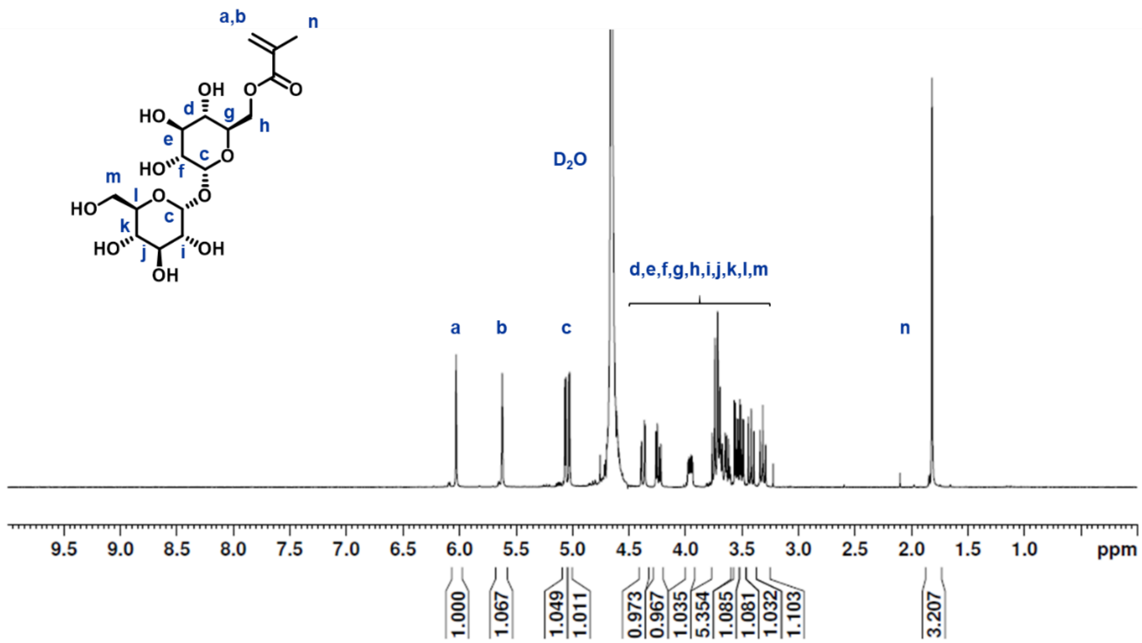

Figure S30. ^{1}H NMR spectrum (400 MHz, D_2O) of trehalose methacrylate (TrMA). ^{1}H-NMR agreed with that reported for this compound.[1]

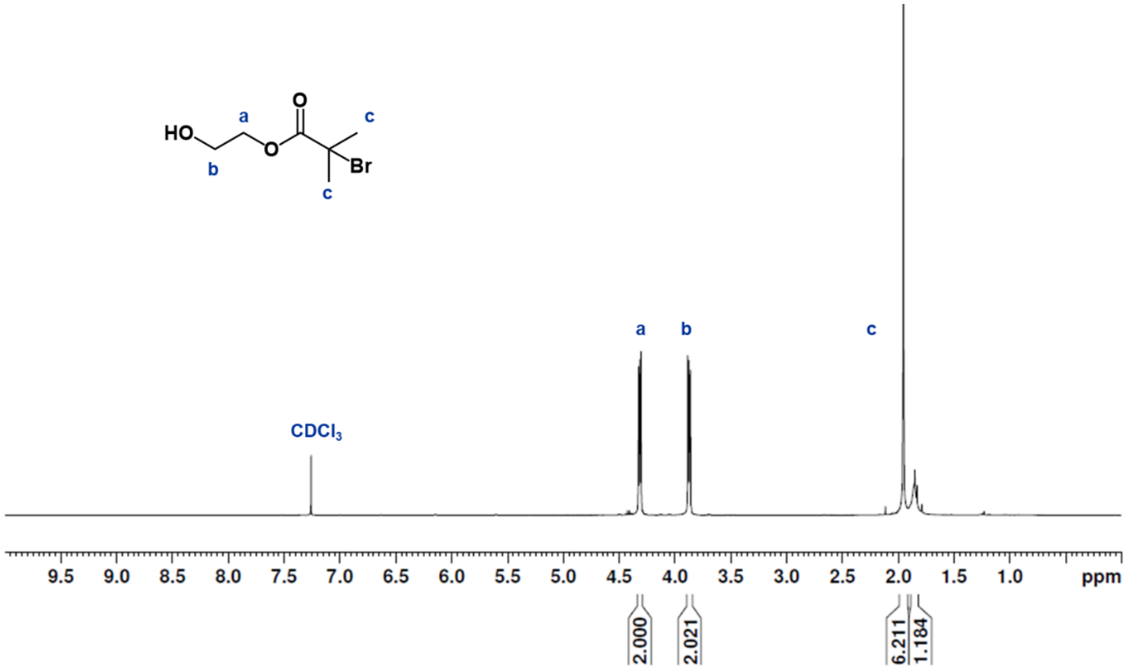

Figure S31. ^{1}H NMR spectrum (400 MHz, $CDCl_3$) of 2-hydroxyethyl 2-bromoisobutyrate (HEBIB) initiator. ^{1}H-NMR agreed with that reported for this compound.[2]

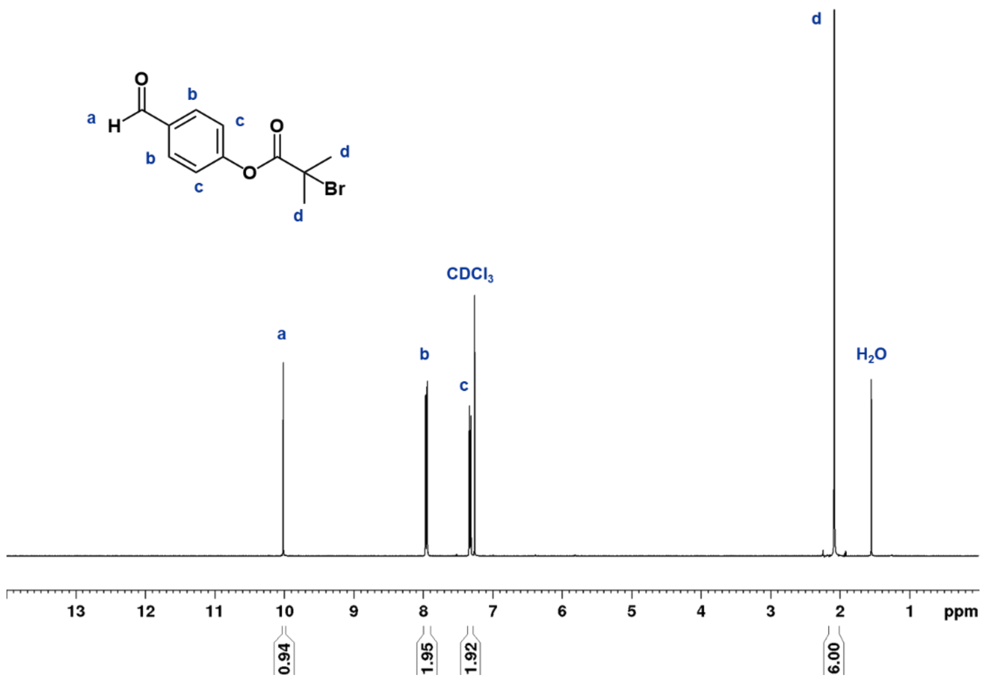

Figure S32. ^{1}H NMR spectrum (400 MHz, $CDCl_3$) of (4-formylphenyl2-bromoisobutyrylate) (benzaldehyde) initiator. ^{1}H-NMR agreed with that reported for this compound.[3]

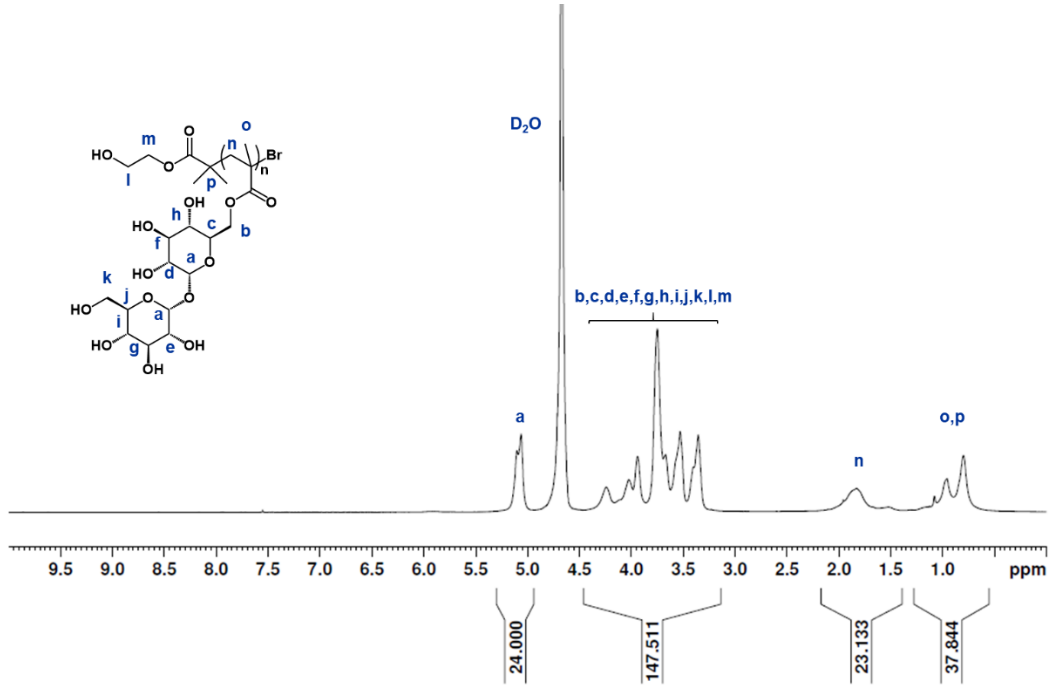

Figure S33. ^{1}H NMR spectrum (400 MHz, D_2O) of pTrMA polymerized with HEBIB initiator. ^{1}H-NMR agreed with that reported for this compound.[2]

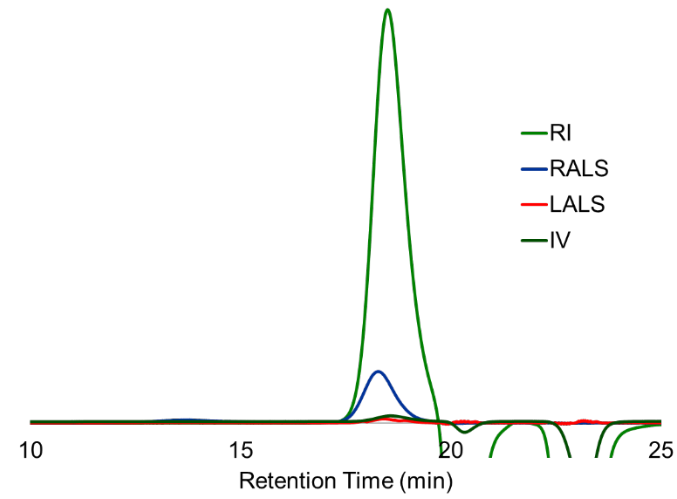

Figure S34. GPC (PEG standards) of pTrMA with HEBIB initiator. M_n = 10.1 kDa, $Đ$ = 1.25.

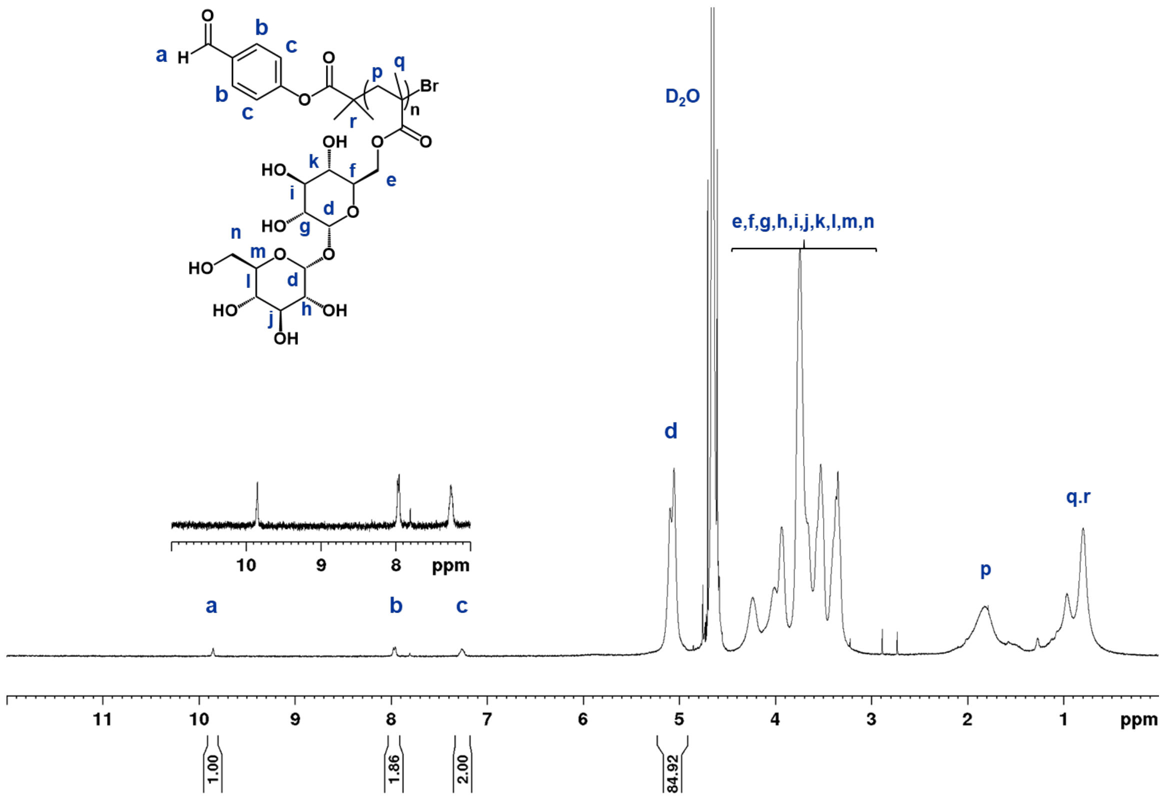

Figure S35. ^{1}H NMR spectrum (400 MHz, D_2O) of pTrMA with benzaldehyde initiator. ^{1}H-NMR agreed with that reported for this compound.[4]

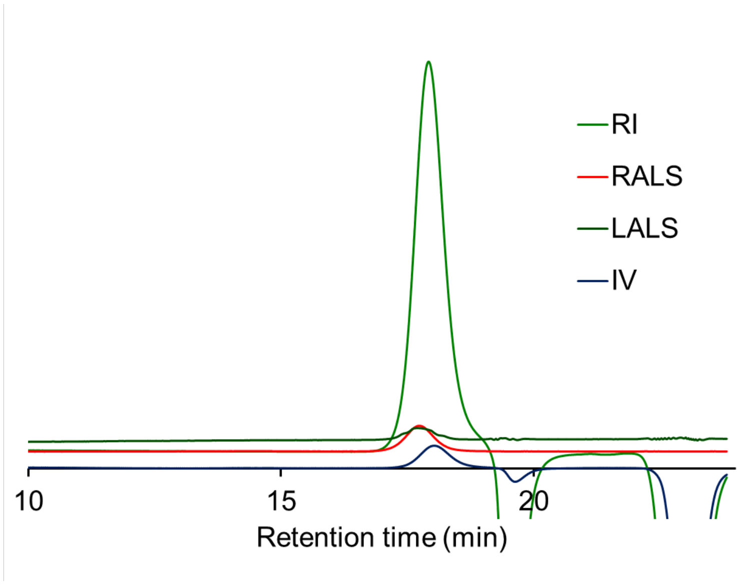

Figure S36. GPC (PEG standards) of pTrMA with benzaldehyde initiator. M_n = 24.9 kDa, Đ = 1.05.

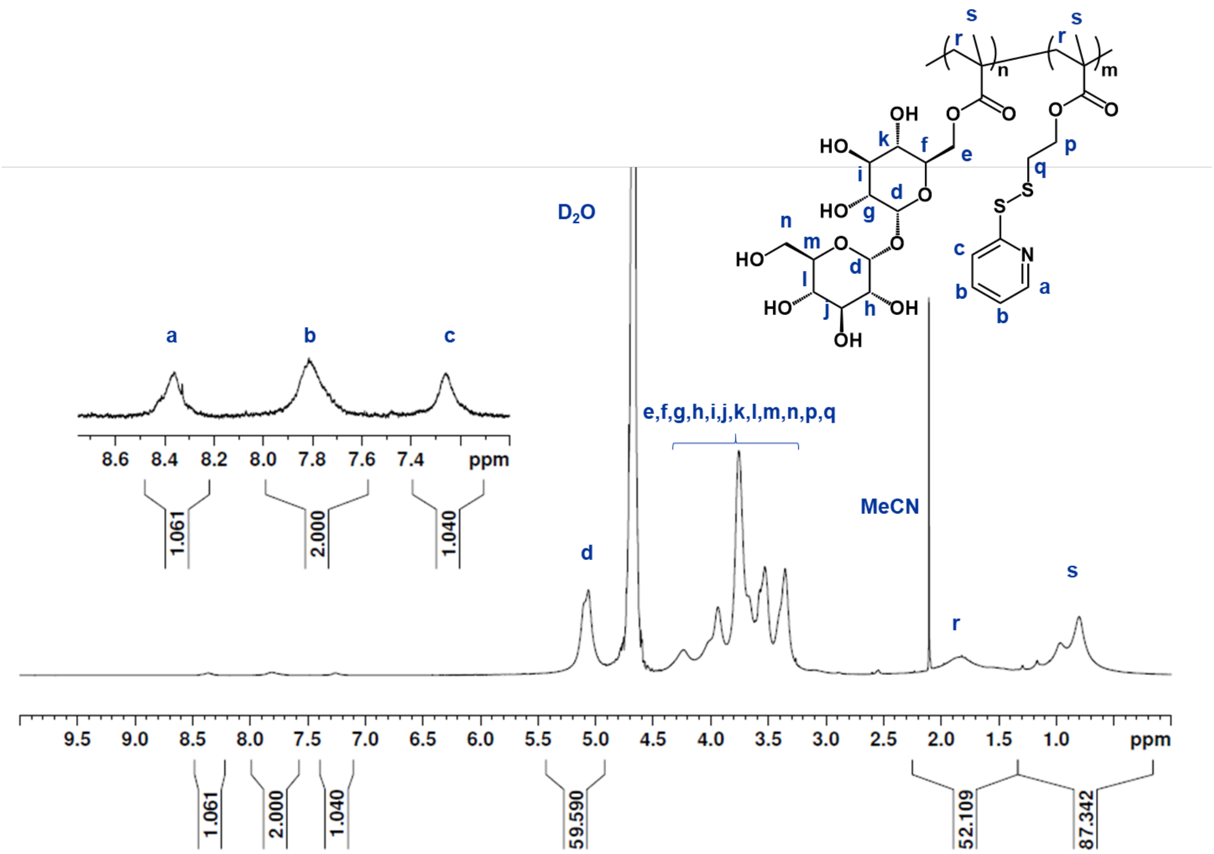

Figure S37. ^{1}H NMR spectrum (500 MHz, D$_2$O) of pTrMA -*co*-PDSMA polymerized by free radical polymerization. Relative PDSMA incorporation was calculated by integration of the PDSMA peaks a, b, and c in comparison with pTrMA peak d. ^{1}H-NMR agreed with that reported for this compound.[5]

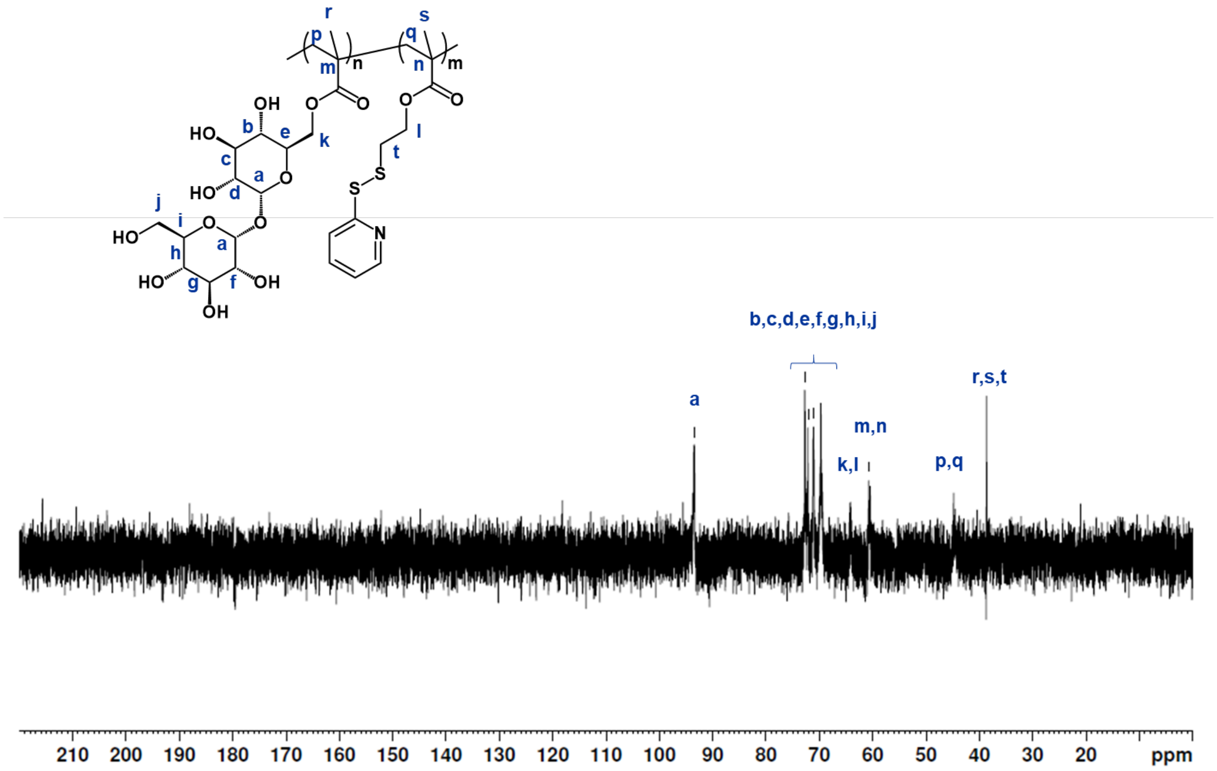

Figure S38. ^{13}C NMR spectrum (500 MHz, D_2O) of pTrMA T-*co*-PDSMA polymerized by free radical polymerization. ^{13}C-NMR agreed with that reported for this compound.[5]

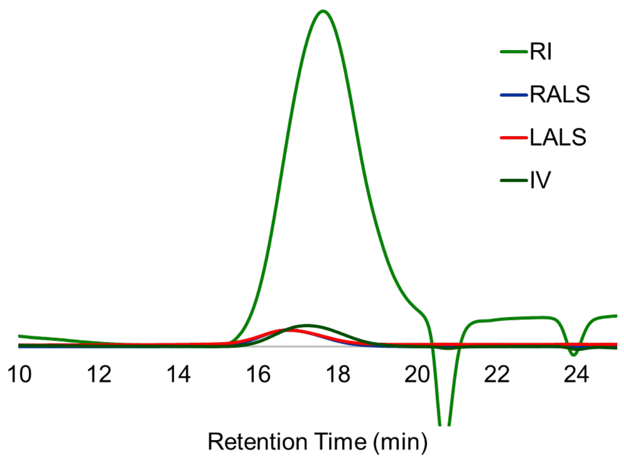

Figure S39. GPC (PEG standards) of pTrMA -*co*-PDSMA. M_n = 8.9 kDa, $Đ$ = 2.19.

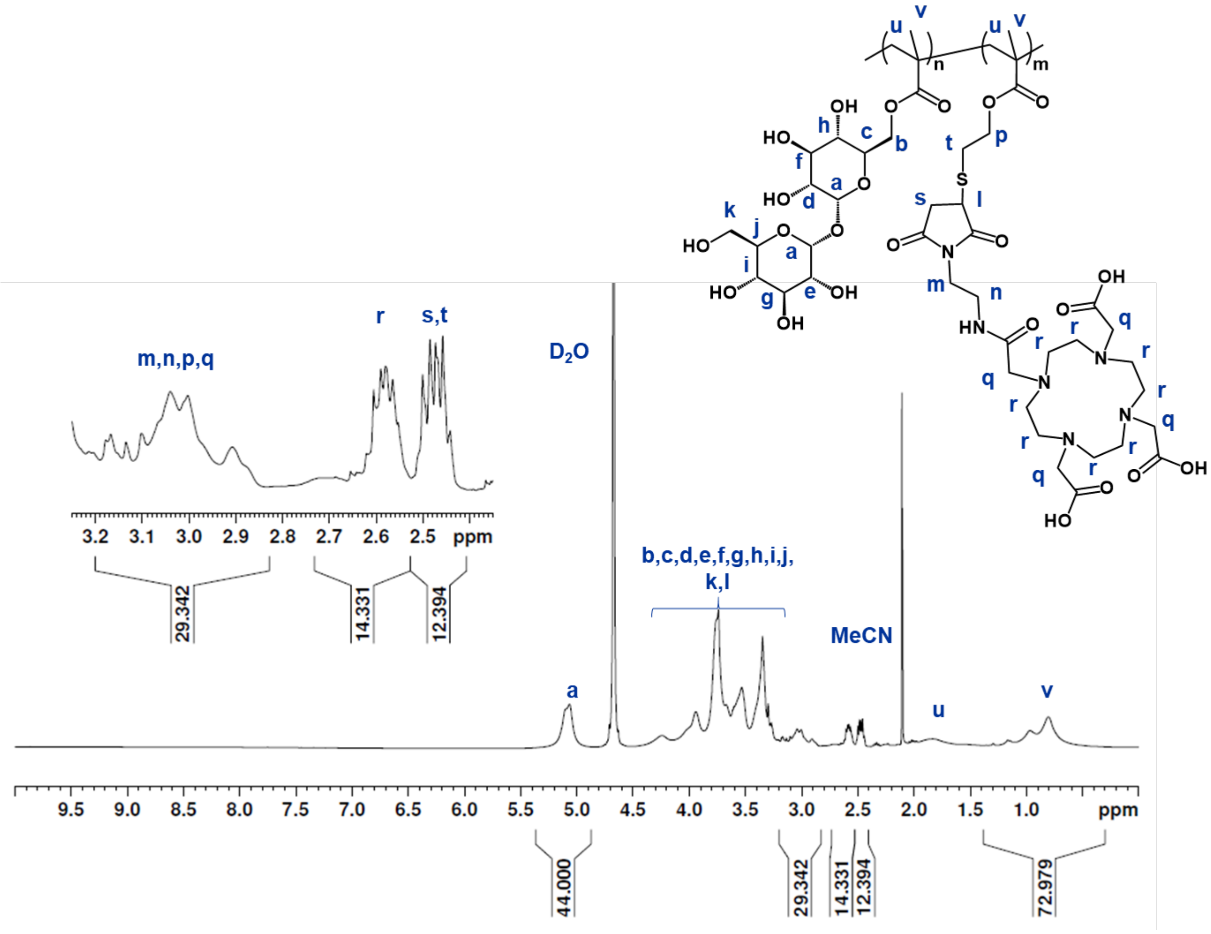

Figure S40. ^{1}H NMR spectrum (500 MHz, D_2O) of pTrMA-*co*-DOTA. Relative DOTA modification was calculated by integration of the DOTA peak r and in comparison with pTrMA peak a.

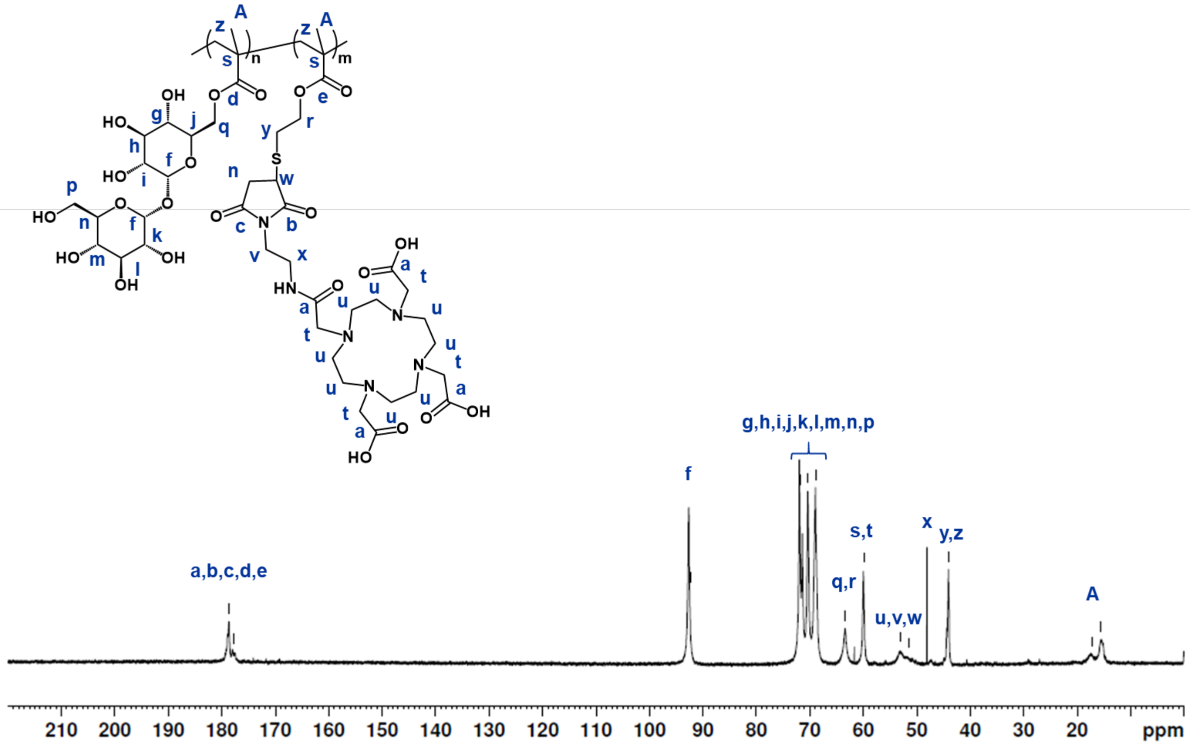

Figure S41. ^{13}C NMR spectrum (500 MHz, D_2O) of pTrMA -*co*-DOTA.

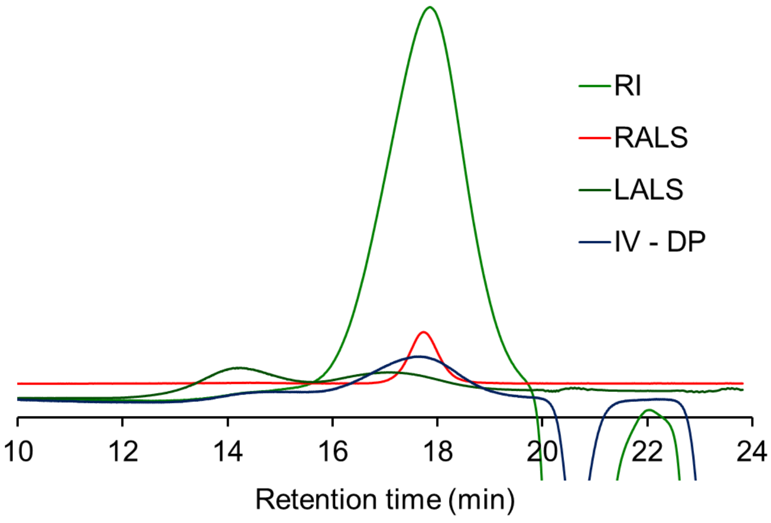

Figure S42. GPC (PEG standards) of pTrMA -*co*-DOTA. M_n = 10.3 kDa, $Đ$ = 2.68.

References

1. Lee, J.; Lin, E. W.; Lau, U. Y.; Hedrick, J. L.; Bat, E.; Maynard, H. D., Trehalose Glycopolymers as Excipients for Protein Stabilization. *Biomacromolecules* **2013,** *14* (8), 2561-2569.
2. Ren, L.; Zhang, J.; Hardy, C. G.; Doxie, D.; Fleming, B.; Tang, C., Preparation of Cobaltocenium-Labeled Polymers by Atom Transfer Radical Polymerization. *Macromolecules* **2012,** *45* (5), 2267-2275.
3. Jin, J.; Liu, J.; Lian, X.; Sun, P.; Zhao, H., Dynamic polymer brushes on the surface of silica particles. *RSC Adv.* **2013,** *3* (19), 7023-7029.
4. Mansfield, K. M.; Maynard, H. D., Site-Specific Insulin-Trehalose Glycopolymer Conjugate by Grafting from Strategy Improves Bioactivity. *ACS Macro Lett.* **2018**, 324-329.
5. Boehnke, N.; Kammeyer, J. K.; Damoiseaux, R.; Maynard, H. D., Stabilization of Glucagon by Trehalose Glycopolymer Nanogels. *Adv. Funct. Mater.* **2018,** *28* (10), 1705475.
